# Effects of turbulent aggregation on clay floc breakup and implications for the oceanic environment

**DOI:** 10.1371/journal.pone.0207809

**Published:** 2018-12-06

**Authors:** Matthew J. Rau, Steven G. Ackleson, Geoffrey B. Smith

**Affiliations:** 1 Mechanical and Nuclear Engineering Department, The Pennsylvania State University, University Park, PA, United States of America; 2 Remote Sensing Division, U.S. Naval Research Laboratory, Washington D.C., United States of America; Delft University of Technology, NETHERLANDS

## Abstract

Understanding how turbulence impacts marine floc formation and breakup is key to predicting particulate carbon transport in the ocean. While floc formation and sinking rate has been studied in the laboratory and *in-situ*, the breakup response to turbulence has attracted less attention. To address this problem, the breakup response of bentonite clay particles flocculated in salt water was studied experimentally. Flocs were grown in a large aggregation tank under unmixed and mixed aggregation conditions and then subjected to turbulent pipe flow. Particle size was quantified using microscope imaging and *in-situ* measurements obtained from standard optical oceanographic instruments; a Sequoia Scientific LISST-100X and two WET Labs ac-9 spectrophotometers. The LISST instrument was found to capture the breakup response of flocs to turbulent energy, though the resulting particle size spectra appear to have underestimated the largest floc lengthscales in the flow while overestimating the abundance of primary particles. Floc breakup and the resulting shift towards smaller particles caused an increase in spectral slope of attenuation as measured by the ac-9 instruments. The Kolmogorov lengthscale was not found to have a limiting effect on floc size in these experiments. While the flocs were found to decrease in overall strength over the course of the two-month experimental time period, repeatable breakup responses to turbulence exposure were observed. Hydrodynamic conditions during floc formation were found to have a large influence on floc strength and breakup response. A non-constant strength exponent was observed for flocs formed with more energetic mixing. Increased turbulence from mixing during aggregation was found to increase floc fractal dimension and apparent density, resulting in a shift in the breakup relationships to higher turbulence dissipation rates. The results suggest that marine particle aggregation and vertical carbon transport concepts should include the turbulence energy responsible for aggregate formation and the resulting impact on floc strength, density, and the disruption potential.

## Introduction

Aggregation is the process in which colloidal particles stick together to form larger particle clusters. This process affects many fields, including wastewater clarification [1], the dredging of navigational channels [2], and even the effectiveness of dry-particle inhaled drug delivery methods [3]. Carbon sequestration within the global ocean is also governed by flocculated particulate matter. The slow sinking of organic flocs from surface waters, often referred to as marine snow, is believed to be a primary carbon sequestration pathway within the global ocean, often referred to as the ocean biological pump [4] and is a key consideration in global climate models [5]. Sinking rates depend heavily on floc size and apparent density, *ρ*_*a*_ kg m^-3^ [6]. Turbulence-induced breakup of marine flocs can reduce their sizes and corresponding settling rates [6,7], thus altering the expected export rate of carbon from energetic surface waters. Thus, understanding how turbulence impacts floc formation and breakup is key to predicting particulate carbon transport in the ocean.

Particles can be brought together by collisions resulting from turbulent motions [8], differential settling velocity [9], or through the trapping of particles within organic matrices [10]. Signatures of floc breakup due to turbulence have been readily observed in the environment, though our current lack of understanding of the breakup processes limits our ability to quantify its importance. Characterization of suspended particulate matter in the ocean is typically performed using non-destructive, *in-situ* optical methods due to the fragile nature of the flocs [11]. Two common methods include spectral light attenuation [7] and near-forward scattering measurements [12]. Spectral attenuation, *c*(*λ*) m^-1^, is often used to measure the concentration of suspended particulate matter (see Nomenclature section for a complete list of symbols, units, and definitions used). In the absence of strong absorption due to dissolved matter, *c* is most sensitive to changes in suspended particulate mass, *X* g m^-3^, and, when referenced to pure water attenuation, is traditionally written as *c*_*p*_ m^-1^. The relationship between *X* and *c*_*p*_ is linear, such that

$$c_{p}=Xc_{p}^{*},$$ (1)

where $c_{p}^{*}$ m^2^ g^-1^ is the mass-specific particle attenuation coefficient.

Particle attenuation generally decreases with increasing wavelength and the spectral dependence can be represented as *c*_*p*_(*λ*) = *Θλ*^-*γ*^. For marine particle populations distributed in size according to a power-law size distribution, the particle size spectrum can be described by *N*(*D*) = *N*_*0*_(*D*/*D*_*0*_)^-*ξ*^, where *D* is particle diameter, and *D*_*o*_ and *N*_*o*_ are reference diameter and concentration, respectively. The slope of the number size distribution, *ξ*, is related to spectral slope of attenuation as *ξ* = *γ* +3 [7]. Thus, a shift in the particle size distribution towards smaller particles (due to breakup) should result in an increase in both *ξ* and *γ*. Boss *et al*. [13] measured *c*_*p*_(*λ*) in the Mid-Atlantic Bight and reported an increase in *γ* simultaneous with the passage of hurricane Edouard. They hypothesized that turbulent energy associated with the hurricane resulted in floc breakup and a shift in the suspended particle population to smaller sizes. Similar trends were reported by Ackleson [14] with measurements of particulate matter in the Long Island Sound and Connecticut River Plume. Higher values of *γ* were observed within the plume boundary, suggesting smaller particles caused by floc breakup in the presence of strong current shear. More-recently, Slade *et al*. [15] have reported how *γ* responds to changes in floc size resulting from turbulence-induced breakup, reformation, and subsequent size-dependent sinking. Observations of Δ*γ* have thus far only been interpreted as relative changes in particle size with no quantitative descriptions as a function of turbulent energy.

To further our understanding of floc breakup dynamics and, by extension, how such processes may impact the fate of suspended marine particles, the breakup response of bentonite clay flocs was studied in a controlled laboratory setting using common optical oceanographic instrumentation. An experimental facility was developed that accommodated the instrumentation while allowing for the precise control of floc formation and exposure to turbulent energy. The optical signatures of floc breakup were observed and compared with floc size and structure determined using optical microscopy. Emphasis was placed on evaluating how hydrodynamic conditions experienced during floc growth affected their overall strength and breakup response. In addition to presenting the results of these experiments, we discuss the implications of these findings to naturally occurring marine particles.

## Aggregation and breakup

### Theoretical background

Accurate prediction of the fate of marine flocs will ultimately rely on robust aggregation and breakup models. These dynamics are typically modeled using the population balance equation (PBE), solutions to which have been thoroughly studied for a range of applications [16–18]

$$\frac{dN(m,t)}{dt}=A_{i}-A_{o}+B_{i}-B_{o}+W+S_{io}$$ (2)

where *N* is the time-varying number distribution of particles of mass *m*. On the right side of the equation, *A* refers to terms that regulate aggregation and *B* refers to terms for floc breakup. The term *W* describes the loss of particulate mass due to gravitational settling, while *S*_*io*_ represents either a source or sink of particulate mass. Aggregation rates are typically given as

$$\begin{array}{l} A_{i}=\frac{\alpha }{2}\int_{0}^{m}\beta (m_{j},m-m_{j})N(m-m_{j},t)N(m_{j},t)dm_{j}\\ A_{o}=\alpha N(m,t)\int_{0}^{\infty }\beta (m,m_{j})N(m_{j},t)dm_{j}. \end{array}$$ (3)

The term *A*_*i*_ describes the rate at which smaller particles aggregate into flocs of mass *m* while *A*_*o*_ describes the rate at which flocs of mass *m* aggregate into flocs of even greater mass. The variable *β* is the aggregation kernel describing how often two particles come into contact based on their size and the surrounding hydrodynamics. The variable *α* describes the probability that particles will adhere to each other, thus forming a floc. Aggregation of a particle population has been studied extensively and well-accepted equations describing *α* and *β* exist in the literature [19–21].

Breakup of particle aggregates has seen less agreement in the literature. Similar to the aggregation terms described above, breakup can be expressed in the PBE as functions of the particle number distribution [22]

$$\begin{array}{l} B_{i}=\int_{m}^{\infty }K(m_{j})\Gamma (m,m_{j})N(m_{j},t)dm_{j}\\ B_{o}=K(m)N(m,t),\\ \mathrm{where}:\;\int_{0}^{m}m_{j}\Gamma \left(m,m_{j}\right)dm_{j}=m \end{array}.$$ (4)

In Eq (4), *B*_*i*_ is the rate at which larger flocs break up into flocs of mass *m* and *B*_*o*_ describes the rate at which flocs of mass *m* break into even smaller particles. Within these terms, *K* is the breakup kernel and defines the frequency that a floc breaks apart. The variable Γ is the mass re-distribution function to describe the size distribution of the breakup mass, which must meet the criteria listed in Eq (4) so that mass is conserved during the breakup process. Currently, no well-accepted or universal definitions for *K* or Γ exist to describe how flocs break apart due to hydrodynamic forcing. Models that have been introduced typically rely on fitting parameters specific to the particles and aggregation system of interest [23].

It is essential to note that floc formation and breakup are caused by the same fluid flow phenomena; turbulent energy and associated fluid motions. Thus, for a given particle population subjected to a defined level of turbulent energy, Eq (2), in the absence of sources and sinks, describes the steady-state particle mass distribution resulting from a balance between floc formation and disruption.

The complex structure and makeup of flocs adds to the modeling complexity. Flocs formed within natural aquatic environments tend to be fractal in structure, where total encased floc volume (*V*_*f*_) and size are related through the volume fractal dimension (*D*_*f3*_) as [24]

$$V_{f}\propto l_{\max}^{D_{f3}},$$ (5)

where *l*_*max*_ is the maximum length of the floc. A fractal dimension of *D*_*f3*_ = 3 indicates a solid particle, while lower dimensionality indicates a porous structure. Fractal dimensions of marine particles vary with reported values between 1.3 and 2.3 [19]. This range of fractal dimension means that two flocs of the same size could have very different internal pore structure and, thus, different breakup strengths. Variations in floc apparent density and structure can be caused by variations in the primary particle material [25] or can result from the formation hydrodynamics during aggregation [26].

An early attempt to develop a turbulence-induced floc breakup relationship was given by Parker *et al*. [27]. In their study, they derived theoretical breakup kernels for the PBE by first determining the floc mechanical strength when exposed to turbulence with a specified rate of fluid shear. Since this study, additional attempts to quantify floc breakup rate have been reported [1,28–31]. However, refinement is needed before predictions regarding the marine environment can be made with confidence.

### Experimental strength determination

A necessary step in the development of a breakup relationship is the determination of floc strength for the particulate matter of interest. Typically, the strength of flocs is quantified through a strength exponent, *n*, obtained from a power-fit to data of the largest floc size plotted against the turbulent energy dissipation rate, *ε*, [32];

$$D_{\max}=C\varepsilon^{-n},$$ (6)

where, *D*_*ma*x_ is the maximum diameter of the floc population, and *C* is the floc strength coefficient. Flocs can break due to bulk fracture or stripping of primary particles from their surface and this strength exponent has been shown to vary with breakup mechanism [27]. The exponent can be linked to the breakup strength by assuming that the strength of the largest floc is balanced by the hydrodynamic breakup stress from the surrounding turbulence, similar to the approach reported by Hinze [33] applied to investigations of bubble breakup in turbulence.

The size of the flocs is important for determining the relevant turbulence forces leading to breakup. Aggregating particles in the ocean are typically small (*D* ~ O^-6^ m) but can grow into flocs ranging from tens of microns to several millimeters in diameter [11]. While the exact mechanism can vary, in all cases the forces at lengthscales similar to the size of the flocs and primary particles is of interest [27]. For large marine snow flocs, these lengthscales can easily reach into the inertial convection subrange of turbulence. In contrast, small inorganic flocs and primary particles in the ocean are typically smaller than the smallest eddies in the flow, defined by the Kolmogorov lengthscale, *η*;

$$\eta ={\left(\frac{\nu^{3}}{\varepsilon }\right)}^{1/4},$$ (7)

where *ν* is the kinematic viscosity. Fluid shear (*G*) at these scales is defined as

$$G=\sqrt{\frac{\varepsilon }{\nu }}.$$ (8)

Floc strength exponents are typically obtained either from plots of maximum diameter plotted against shear or directly plotted against the dissipation rate as indicated in Eq (6) [32].

Strength exponents reported for flocs of clay particles similar to those used in this study are summarized in Table 1. Also included are values obtained by [34] for naturally-sampled marine snow particles for comparison. According to the theory presented by Parker *et al*. [27], flocs in either the inertial convection subrange of turbulence or the viscous dissipation subrange should have strength exponents of *n* ≈ 0.25. Many of the results from the literature show values close to this theoretical value; however, there is significant spread in the data. It should be noted that all results for clay presented in Table 1 were obtained using turbulence created with mixing impellers in jar tests, where determining a representative value of dissipation rate is difficult due to the inherent non-uniformity in turbulence. Additionally, none of the previous investigations attempt to quantify the effect of turbulent conditions during floc formation on their structure and resulting breakup strength.

**Table 1:** Strength exponents for clay flocs from the literature.

Reference	Type of floc and flocculant	*n*
Hsu and Glasgow (1983) [1]	Kaolinite + polyacrylamide	0.314
Francois (1987) [35]	Kaolinite + aluminum hydroxide	0.15 to 0.75
Tambo and Hozumi (1979) [36]	Kaolinite + aluminum hydroxide	0.33 to 0.38
Bouyer *et al*. (2001) [37]	Bentonite + aluminum	0.33
Li, *et al*. (2007) [38]	Kaolinite + aluminum sulfate	0.19
Zhu, *et al*. (2016) [39]	Kaolinite + NaCl	0.46 to 0.58
Alldredge, *et al*. (1990) [34]	Natural marine snow flocs	0.11 to 0.29
*This study*	Bentonite + NaCl	0.25 to 0.34

## Methods

### Facility

**Fig 1:**
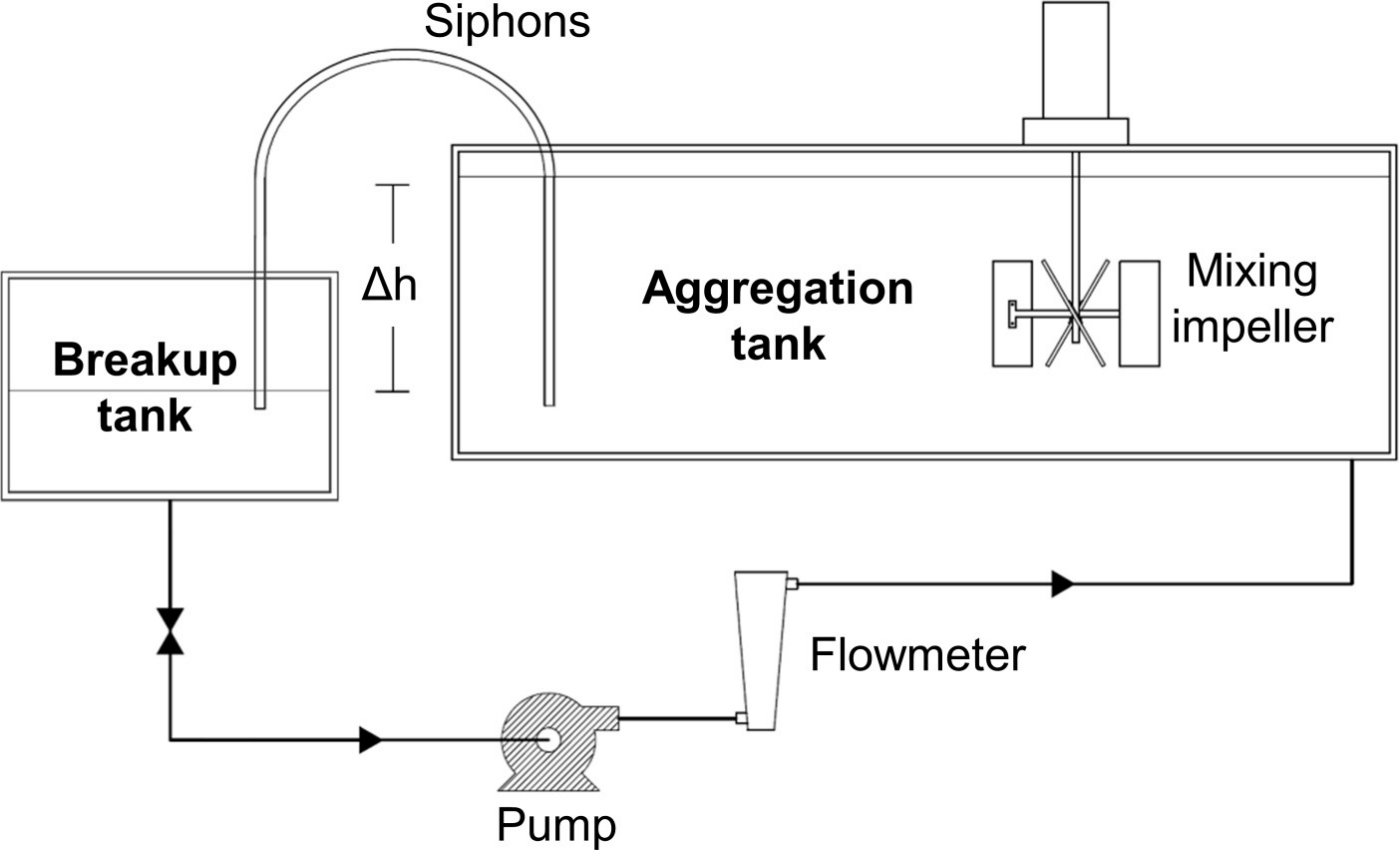
Schematic diagram of the floc breakup facility.

A recirculation loop consisting of two tanks, a larger one in which to generate flocs and a smaller one to receive disrupted particles, was used to investigate the response of Bentonite clay particles to turbulence (Fig 1). The tanks were filled with saline water (10 psu) created with filtered tap water and dissolved sea salt (Instant Ocean). The Bentonite particles (volumetric mean diameter of 3.7 μm, Alfa Aesar, stock no. A15795) were first dispersed in small batches of deionized water through vigorous mixing before being added to the flow facility to reach an overall mass concentration of 40 mg/L. The water mixture recirculated at a constant volumetric flow rate of (9.5 ± 0.26 L/min). Within the large aggregation tank particles were subjected to gentle fluid motion caused by recirculation. In a subset of experiments a mixing impeller was added to the large tank to increase turbulence during floc formation.

Flocs formed in the aggregation tank were subjected to a range of turbulent energy by flow through one or more siphon tubes that connected the large aggregation tank with a smaller tank, referred to as the breakup tank. The siphon tubes had an inside diameter of *d* = 12.7 mm and length *l* = 170*d*. The flow rate was maintained by adjusting the hydrostatic head created by the difference in water level, Δ*h*, between the two tanks. Between one and eight siphon tubes were used to create eight different flow conditions representing either fully-developed laminar or turbulent pipe flow of varying strength. Flow conditions were chosen to span and exceed the range of turbulence dissipation rates reported for the upper ocean, where *ε* can range from 10^−10^ to 10^−1^ m^2^/s^3^ [40] (Table 2). Sample water was returned from the breakup tank to the aggregation tank with a pump. It was found that the pump caused nearly complete floc breakup and the size distribution of the particulate material returning to the aggregation tank was close to that of the primary, unaggregated particles.

**Table 2:** Siphon flow conditions used in the floc breakup experiments.

Number of siphons	Re	*ε* (m^2^-s^-3^)	*η* (μm)	*G* (s^-1^)
8	1947	-	-	-
7	2225	0.010	101	100
6	2596	0.016	91	124
5	3115	0.026	80	159
4	3894	0.048	68	216
3	5192	0.105	56	321
2	7788	0.320	43	561
1	15576	2.152	26	1456

Average dissipation rates of turbulent kinetic energy, *ε*, in the siphon tubes were obtained using the average flow velocity, $\bar{u}$, following Bakewell and Lumley [41], where

$$\varepsilon =\frac{\tau_{w}\pi dl\bar{u}}{\rho \pi ld^{2}/4}.$$ (9)

Wall shear stress, *τ*_*w*_, was obtained assuming fully-developed turbulent pipe flow [42].

The volume of water within the aggregation tank was approximately 550 L and required one hour to recirculate given the constant facility flow rate. The mixing times of the water entering the aggregation tank with and without stirring were estimated from dye visualization to be one minute and two minutes, respectively. This suggests that the particle population within the aggregation tank was well-mixed and consisted of recirculated primary particles and flocs that had been forming for up to one hour.

The breakup tank (0.1 m^3^ capacity) was sized large enough to accommodate the *in-situ* optical sensors, but small enough to maintain a relatively short sample resonance time (~10 min), thus minimizing floc reformation from the disrupted particles. Water volume within the breakup tank was maintained constant by raising and lowering the tank with a hydraulic lift as the number of siphon tubes was changed.

### Instrumentation

Floc size was characterized using *in-situ* measurements of the near-forward particle scattering function, *β*_*f*_*(θ)* and *c*_*p*_(*λ*) and through microscopic imaging of small water samples. Measurements of *β*_*f*_*(θ)* were obtained using a LISST-100X (Sequoia Scientific), which measures near-forward light scatter between 0.08^o^ and 15^o^ [12]. Manufacturer software accompanying the instrument was used to compute integrated particle volume in 32 logarithmically-spaced size bins between 1.86 μm and 187 μm based on assumptions of randomly shaped and oriented particles [43]. The sensor end of the instrument was positioned within each tank to obtain measurements representing each flow condition. The particle volume output by the LISST was the cumulative volume of all particles in each size bin, making the measured spectra a histogram with the volume concentration dependent on the bin width. To generalize the particle spectra, the volume measurements were normalized with the width of each size bin for presentation. In addition to volume spectra, the LISST also provides a measure of the water attenuation at its laser wavelength of 670 nm (*c*_*p*,*LISST*_).

Spectral attenuation was measured with two 9-channel, 25 cm pathlength attenuation meters (ac-9, WET Labs) referenced to clean water; one positioned in each of the two tanks and both operating at wavelengths of 412, 440, 448, 510, 532, 555, 650, 676, and 715 nm. The instruments are designed to measure attenuation of all water impurities within a sample stream pumped through a tube with highly absorbing internal walls. To avoid inadvertent floc breakup caused by turbulence within the sample stream, the tubes were removed and the sensors exposed to the bulk ambient water in each tank. Due to the small acceptance angle of the attenuation sensor (0.93°) and the large water volume in both tanks, it is unlikely that light scattered away from the instrument was reflected or scattered back into the instrument sensor. Attenuation measurements were obtained with the laboratory lights turned off to avoid ambient light detection. All instruments were cleaned and calibrated prior to experimentation using purified, deionized water in accordance with community protocols [44].

Since the experimental clay mixture did not contain any strongly absorbing dissolved material, the measured attenuation is assumed to represent only suspended clay particles and, thus, *c*_*p*_. The spectral slope of *c*_*p*_ was computed using measurements at 532 nm and 650 nm following [14]:

$$\gamma =-\log\left[\frac{c_{p,532}}{c_{p,650}}\right]/\log\left[\frac{\lambda_{532}}{\lambda_{650}}\right].$$ (10)

**Fig 2:**
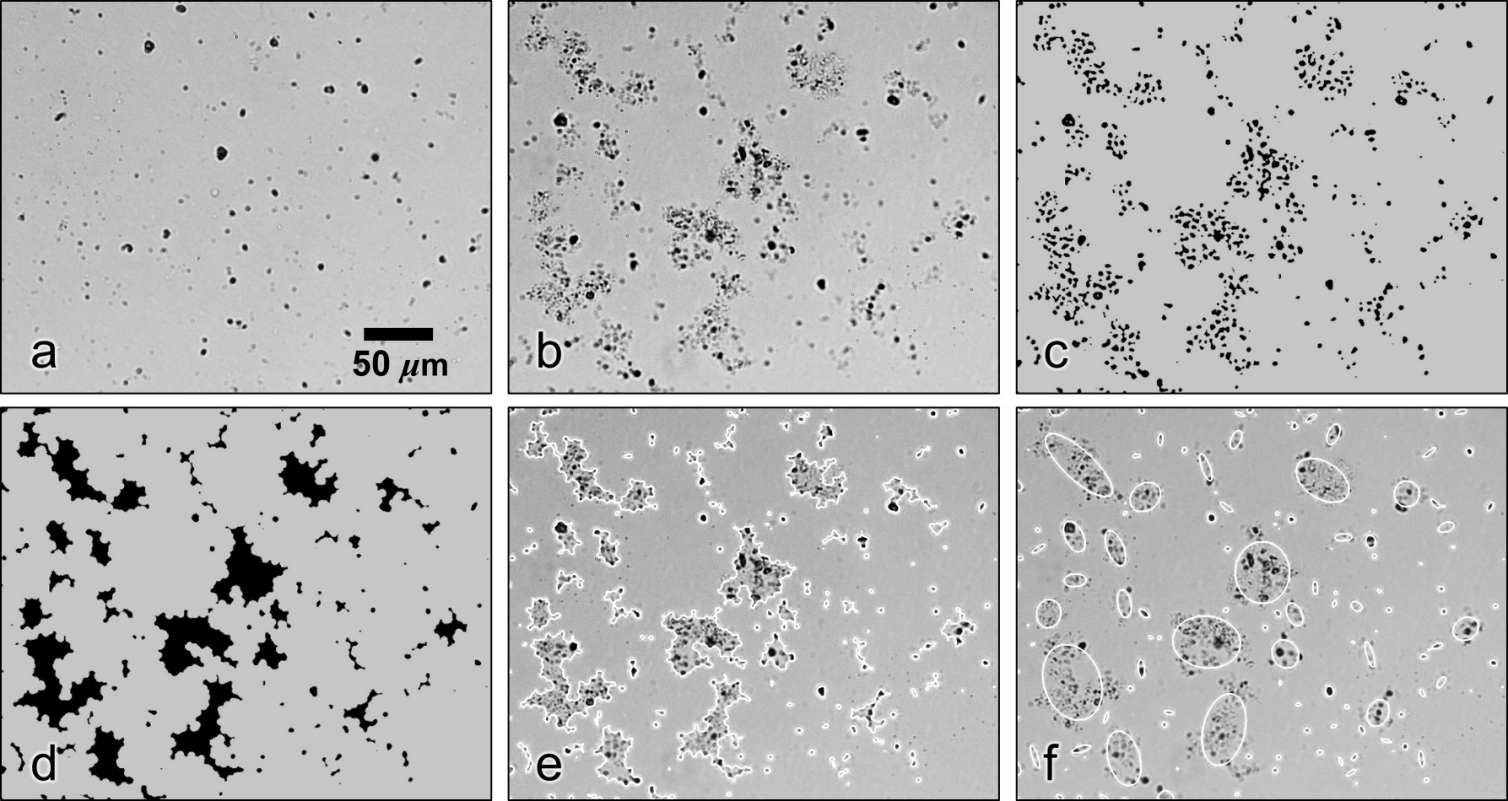
Microscope images of the particles and processing steps used to derive particle measurements. (a) Primary particles flowing from the pump, (b) flocs formed in the aggregation tank, (c) floc image after the thresholding step, (d) final binary image for particle processing (background shading in c and d added for uniformity), (e) perimeters of each floc and (f) fitted ellipses from the imageJ particle analysis overlaid on the microscope image. Scaling of all images is the same.

More detailed analyses of the floc size, shape, and structure were conducted through microscope imaging using an inverted microscope with a 10X magnification objective (Axiovert, Zeiss) (Fig 2). An inverted 10 ml pipette was used to draw a small volume of water from the aggregation tank, the breakup tank, and from the water leaving the pump. The water sample was slowly deposited into a small transparent dish for microscopic examination. This sampling method causes minimal breakup of fragile flocs suspended in the water [28]. One hundred images of each sample were obtained with a 16-megapixel digital camera (D4, Nikon). The overall imaging system resolution was pixel-size limited to 0.29 μm/px.

Each set of images was processed using ImageJ [45] to quantify floc morphology. First, a background image obtained in the absence of particles was subtracted from the raw microscope images. The mean image intensity was then subtracted followed by a mean filter and Mexican-hat filter applied with a 5 px radius. The images were then inverted and thresholded. The resulting binary images (*e*.*g*., Fig 2C), display flocs as groups of primary particles located close to one another but not touching. To obtain a projection of each floc, particles in the image were first dilated by 9 px with a circular element, holes in the resulting features were then filled and the floc images were finally eroded with a 9 px circular element. It was found that these three steps reliably conjoined the images of particles within each floc without erroneously linking separate flocs or altering the final floc dimensions (Fig 2D). Example floc perimeters overlaid on the example microscope image are also presented (Fig 2E). Floc perimeters and additional size statistics were obtained using the Analyze Particles function available in ImageJ.

### Experimental procedure

Experiments were performed by installing the optical instruments and desired number of siphon tubes in the tanks. The clay material was suspended and allowed to recirculate and stabilize for two hours. It was found that the facility recirculation was enough to maintain particle suspension during experimentation and that re-suspension was only necessary at the beginning of each experiment. For tests with stirred aggregation, the mixing impeller rotated at a rate of 25 rpm. Each experiment was performed on a separate day over the course of two months, with unmixed and mixed aggregation tests performed in an alternating fashion. The timeline for data acquisition is provided in Table 3.

**Table 3:** Summary of experimental tests with floc population stability and resulting strength exponents.

			Experiment stability					
Tank condition	Test date (2017)	Plot symbol	${\bar{c}}_{p,LISST}$ (m^-1^)	Δ*c*_*p*,*LISST*_ (%)	${\bar{D}}_{95,LISST}$ (*μ*m)	Δ*D*_*95*,*LISST*_ (%)	*C*	*n*
Unmixed	Feb. 17^th^	◄	11.60 ± 0.17	3.56	162.4	10.1	30.81 ± 1.31	0.33 ± 0.12
	Feb. 23^rd^	●	12.15 ± 0.14	1.87	55.4	5.3	14.25 ± 1.37	0.26 ± 0.04
	Mar. 2^nd^	▲	12.42 ± 0.13	2.66	44.0	20.1	9.86 ± 1.59	0.29 ± 0.05
	Apr. 18^th^	♦	11.76 ± 0.14	2.58	50.3	22.2	13.37 ± 1.64	0.26 ± 0.08
Mixed	Feb. 22^nd^	⬠	13.34 ± 0.05	3.31	39.2	13.3	-	-
	Feb. 28^th^	**▽**	13.16 ± 0.04	1.70	28.1	18.7	-	-
	Mar. 7^th^	**▷**	13.60 ± 0.05	2.73	21.9	24.5	-	-
	Apr. 27^th^	□	12.48 ± 0.05	4.34	18.4	20.3	-	-

Once the optical signals in the tanks were stable, the temperature and salinity of the water was recorded and four minutes of LISST measurements (sampled at 1 Hz) were obtained in the aggregation tank, breakup tank, and in a sample of water leaving the pump. During two of the eight experiments presented, four minutes of attenuation measurements in each tank were also collected directly following acquisition of the LISST measurements. Data from each time series were averaged for analysis.

For each flow condition, a 10 ml water sample was collected from the breakup tank as described above. Images of this water sample were acquired within five minutes of the collection time to minimize changes in the floc size and structure. Once the water sample for microscope analysis was obtained, the number of siphon tubes was changed and data for the next flow condition was acquired following a 40 min stabilization period.

## Results

### Experimental conditions

**Fig 3:**
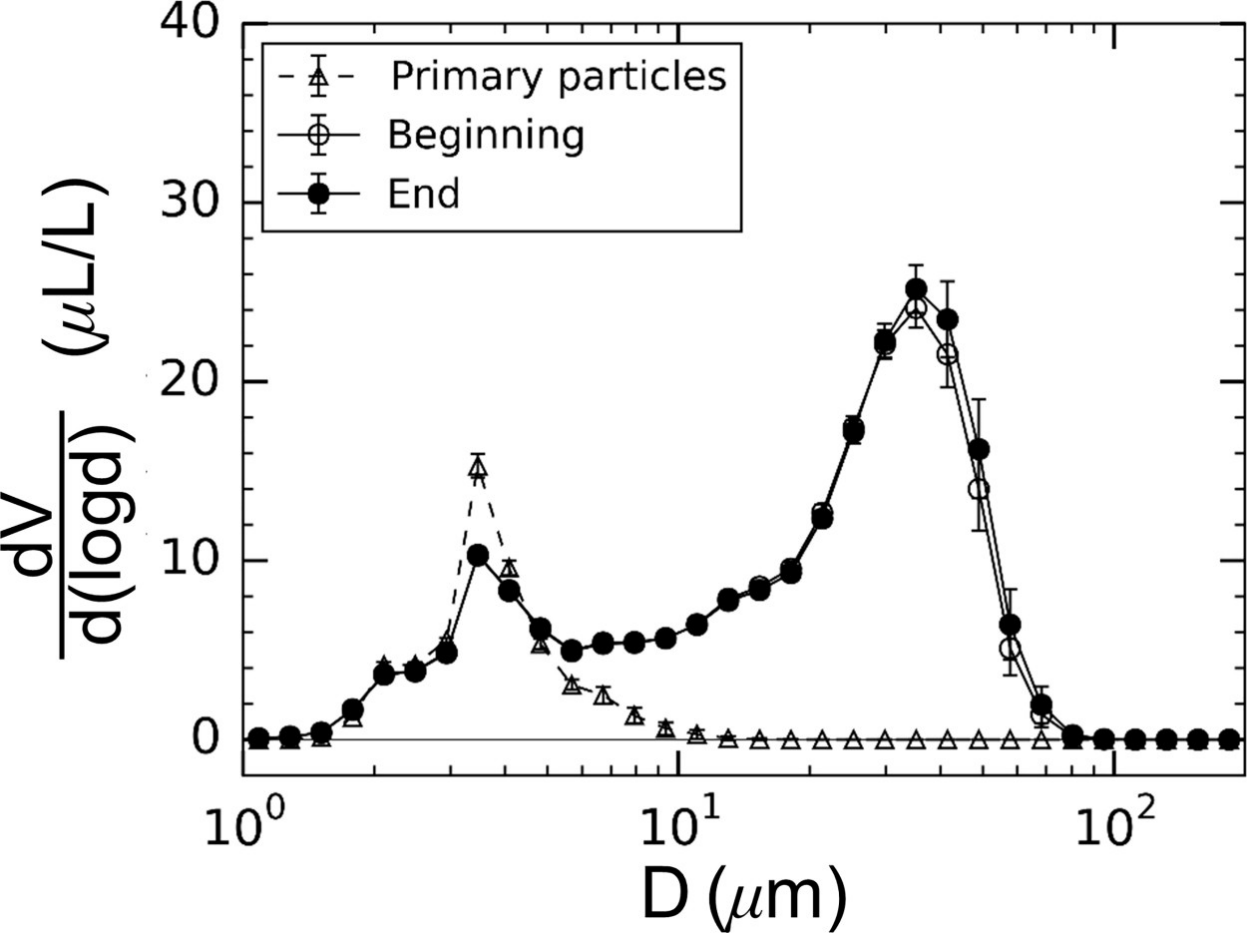
Size-spectra of particle volume for the primary particles and flocs in the aggregation tank measured with the LISST-100X. Consistency of floc size distribution during experimentation is shown with measurements obtained nine hours apart during continuous facility operation. Error bars represent one standard deviation.

The floc population within the large tank was found to be stable over the course of each experiment. Fig 3 shows the floc size distribution measured with the LISST in the aggregation tank at the beginning and end of an experimental run (approximately 9 hours apart). The loss of particle mass due to settling was quantified using the mass-specific particle attenuation coefficient described by Eq (1). The ac9 spectrophotometer response was carefully calibrated with known concentrations of bentonite powder in water to determine a coefficient of $c_{p}^{*}$ = 0.309. For the experiments where the ac-9 was deployed, this provided a direct measure of the bentonite mass concentration. For the mixed aggregation tank, the mass concentration at the beginning of the experiment was 39.4 mg/L compared with 38.7 mg/L at the end, representing a loss of only 0.7 mg/L (1.8%) due to settling. Without the mixing impeller, the mass concentration decreased by a similar percentage from 36.8 mg/L to 36.1 mg/L (a change of 1.9%). The lower mass concentrations measured for the unmixed case were likely due to the largest flocs having settled to the bottom of the tank during the initial stabilization period. The change in *γ* in the aggregation tank was also monitored and found to change by 1.2% and 0.8% for the mixed and unmixed experiments, respectively. Because the ac-9 was not deployed in every experimental run, the attenuation measurements from the LISST were also monitored as were the changes in the 95% percentile particle diameter, *D*_*95*_, calculated from the particle spectra. A summary of the change in these measurements is provided in Table 3.

**Fig 4:**
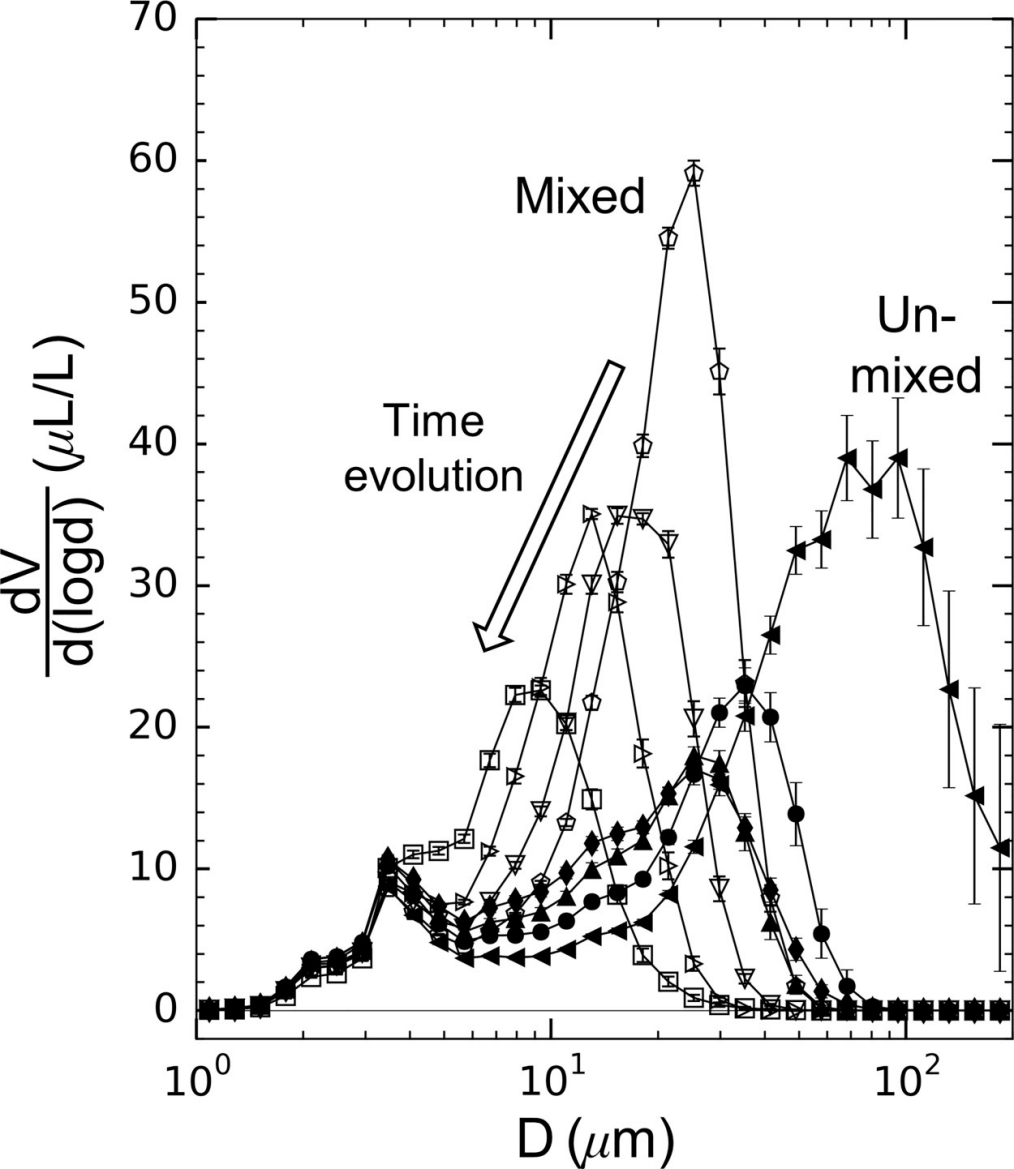
Particle size spectra measured in the aggregation tank for all eight experimental runs. Symbols consistent with Table 3. Error bars represent one standard deviation.

Experiments were performed using the same solution of particles and salt water over the course of two months. Slow evolution of the steady-state particle spectra occurred during this time. Shown in Fig 4 are the particle size spectra measured in the aggregation tank for all experimental cases. For both the unmixed and mixed tank conditions, the floc sizes decreased as the experiments progressed. This evolution occurred despite maintaining consistent tank and flow conditions and carefully following the experimental procedure outlined above. It is also apparent in Fig 4 that mixing the aggregation tank resulted in compressed particle spectra with higher relative volumes of flocs but at smaller floc diameters. Despite this slow evolution of particle spectra, the flocs grown showed repeatable breakup trends, as described below.

### Characterization of changes in floc size

#### LISST measurements and particle lengthscales

Floc formation and breakup was identified by observing changes in the measured particle sizes with turbulence exposure. By measuring particle size spectrum, various size characteristics of the population could also be determined (*e*.*g*. volumetrically-weighted mean diameter, *D*_*vol*_, or the 95^th^ percentile diameter, *D*_*95*_). Representative spectra for the primary particles and flocs formed in the unmixed aggregation tank are shown in Fig 3. In the experiments, the primary particle size distribution had a strong, primary peak particle volume at 3.5 μm. Within the aggregation tank, in contrast, the particle sizes exhibited a bi-modal distribution with peaks at 3.5 μm and 35 μm for the results presented (Feb. 23^rd^, 2017). Since particle mass during each experiment was stable, as discussed above, the large increase in volume at larger particle sizes is indicative of floc formation. The LISST measurements best approximate the total encased volume of flocs, which means that any increase in floc volume without additional particulate mass indicates a decrease in fractal dimension (Eq 5).

The light scattered to each ring of the LISST-100X detector is primarily dependent on the area of the particle projection within the laser beam. Because of this and the fact that particles are randomly oriented within the water volume, the sizes from the LISST best represent an average of the particles as viewed from many orientations rather than a true measure of particle lengthscales. To test the accuracy of the LISST spectra compared to the actual floc lengthscales, measurements were compared to sizes obtained from the microscope images described above. Particle sizes were defined using the minor (*D*_*minor*_) and major (*D*_*major*_) axes of ellipses fit to the floc images and the maximum Feret diameter, which is a measure of the maximum possible length through the floc (*l*_*max*_). These size statistics were obtained for all particles in the images for each flow condition, typically including ~300,000 flocs, and then distributed into size bins equivalent to those of the LISST to create the size spectra. Particle volume was calculated for each floc by multiplying its projected area with the root-mean-square of the fitted ellipse minor and major axes as an estimate of the out-of-plane floc dimension.

**Fig 5:**
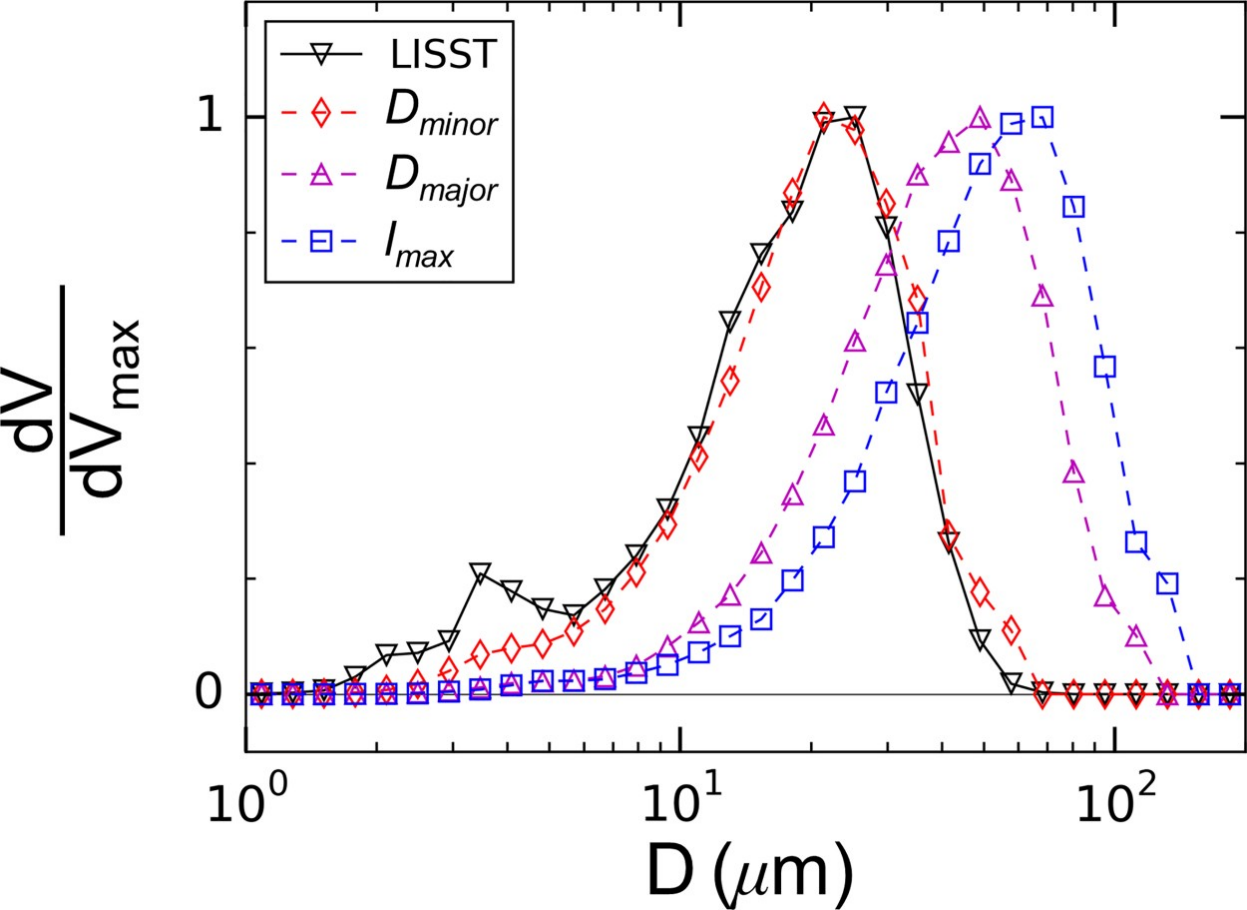
Normalized volume spectra measured with the LISST compared with spectra obtained from the microscope images.

Fig 5 shows an example comparison of spectra for flocs in the aggregation tank. For comparison with the LISST measurements, all spectra were normalized by the maximum particle volume. Agreement in volume concentration between the two measurement methods was not expected given that sampled particles settled out of suspension onto the bottom of the microscope dish for imaging. The size spectra created using the ellipse minor axes (*D*_*minor*_) were in close agreement with the LISST measurements (Fig 5). The microscope measurements did not capture the relative volume concentration of small particles (*D* < 15 μm) observed in the LISST measurements, but the overall shape of the spectra were in good agreement. Given that the particle resolution of the microscope imaging system was 0.29 μm, the disparity for small particles cannot be explained by insufficient image resolution. One explanation for the discrepancy is that particles on the bottom of the microscope dish likely landed broad-sided, meaning that the microscope did not sample the smallest dimension of the smallest particles. A second and perhaps compounding explanation is suggested by Graham *et al*. [46], who provided evidence that the scattering signal measured by the LISST-100X may include aspects of both the small and large particle lengthscales, meaning that the LISST may overestimate the volume of primary particles in flocculated suspensions with resulting size spectra that are some combination of both the flocs and the constituent particles that comprise them. While it will be shown in a later section that the LISST spectra can accurately capture the floc breakup response, the microscope measurements are also considered to fully evaluate the relevance of the largest lengthscales of the flocs.

#### Spectral slope of attenuation

**Fig 6:**
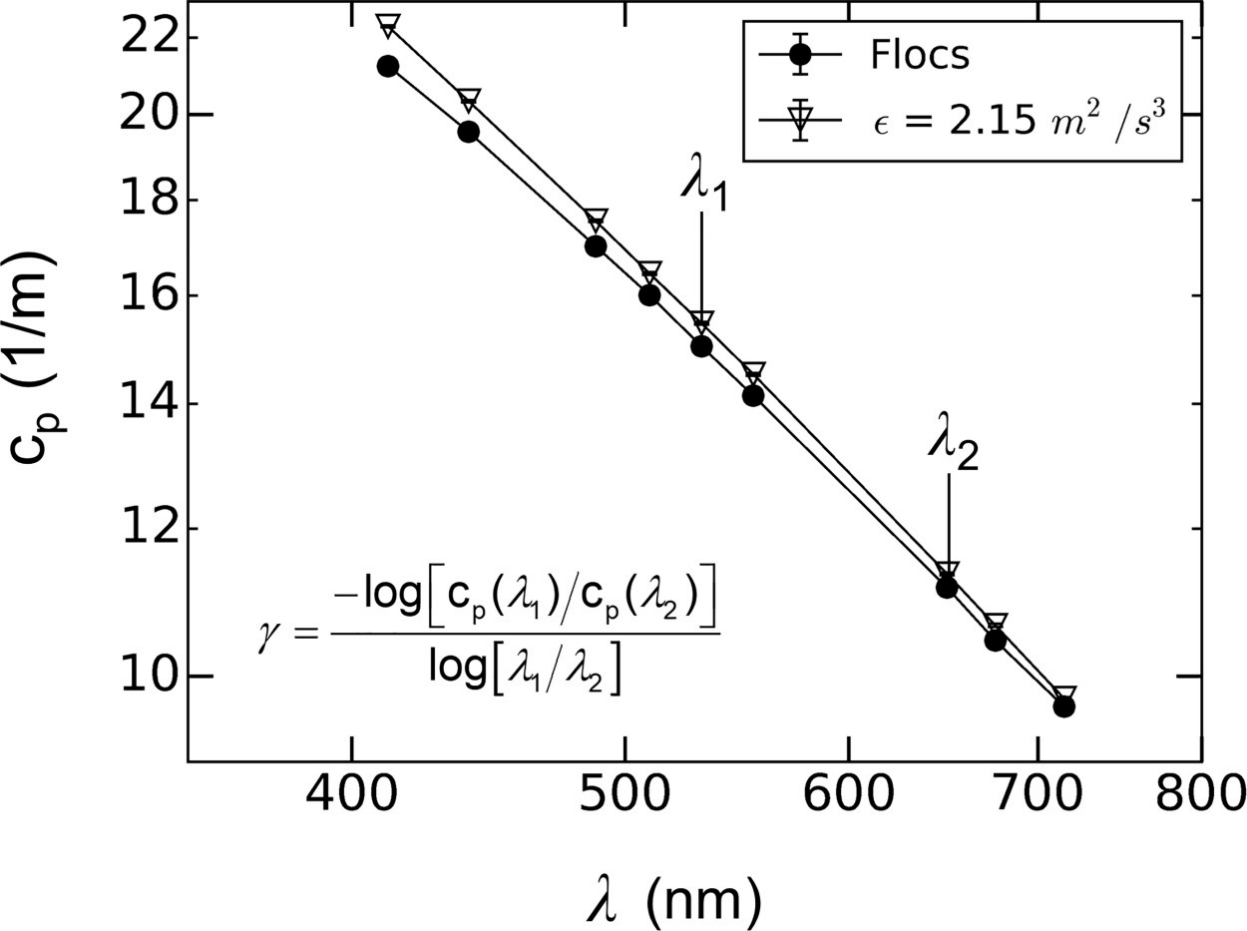
Plot of in-water light attenuation measured with the ac-9 spectrophotometer as a function of wavelength.

The slope of spectral light attenuation (*γ*) was calculated according to Eq (10). Plotted in Fig 6A are representative attenuation measurements collected on April 18^th^, 2017 at all nine wavelengths obtained for flocs in the unmixed aggregation tank and the same particle population after flowing through the siphon tubes with a dissipation rate of 2.15 m^2^/s^3^. The associated change in *γ* as a function of turbulence dissipation rate are presented in Fig 6B. While the changes in attenuation signal between these two states were small, the spectral slope increased with turbulence indicating an associated shift in particle size towards smaller particles. These results are discussed in more detail in the following section.

### The effect of formation dynamics on floc disruption characteristics Changes in floc sizes due to turbulent flow

**Fig 7:**
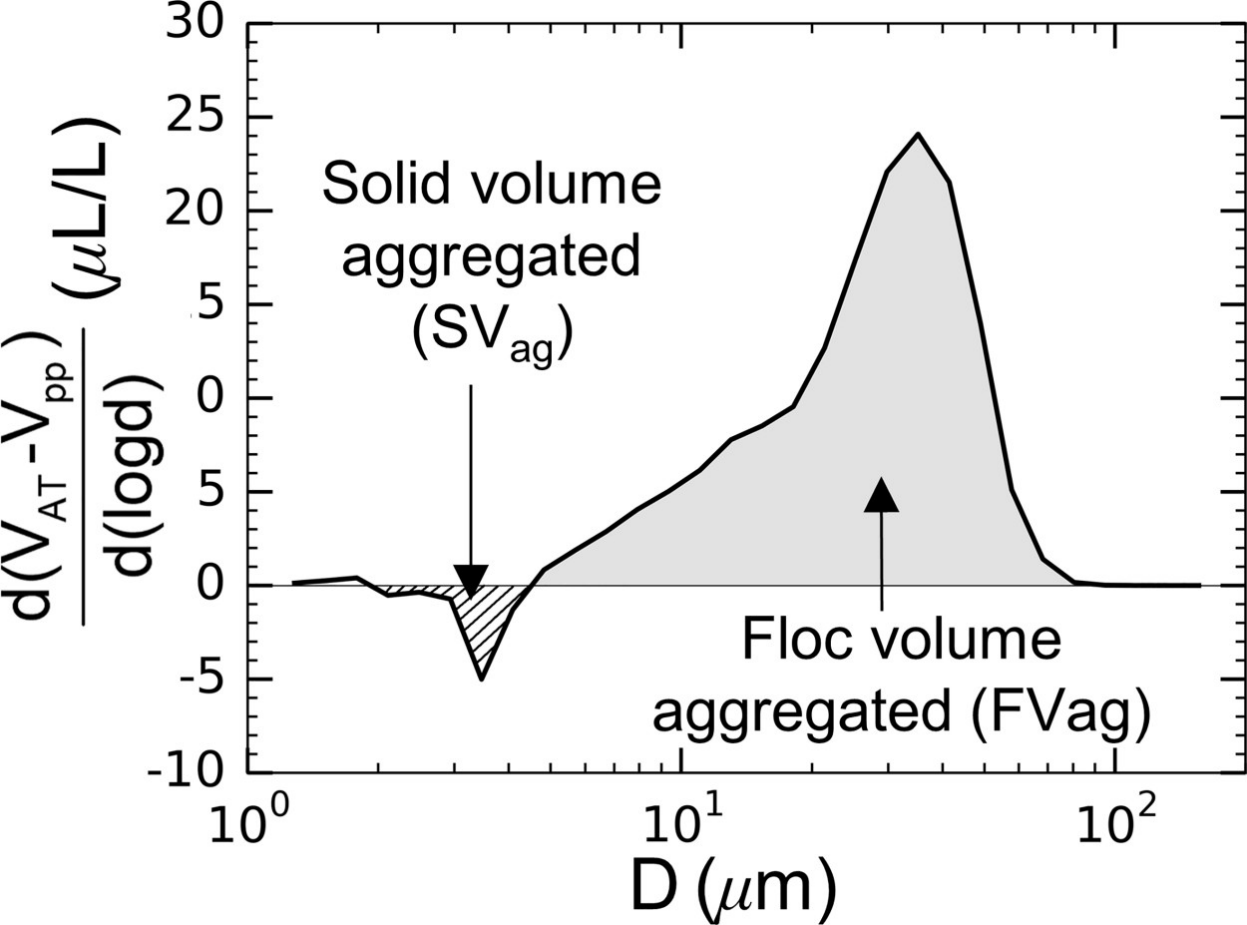
The difference in particle volume between the aggregation tank and the primary particle distribution. This figure illustrates the calculation of the volume of primary particles removed from the population (*SV*_*ag*_) and the floc volume formed due to aggregation (*FV*_*ag*_).

Unmixed and mixed aggregation tank conditions were used to create two distinct floc populations. A summary of floc characteristics for both the unmixed and mixed conditions are summarized in Table 4. Here, the volume of aggregating primary particles, *SV*_*ag*_, and flocs created due to aggregation, *FV*_*ag*_, are compared. Their respective total volumes were calculated from the LISST spectra as illustrated in Fig 7 with,

$$SV_{ag}=\left|\sum_{if<0}\left(V_{AT}-V_{pp}\right)\right|$$ (11)

$$FV_{ag}=\sum_{if>0}\left(V_{AT}-V_{pp}\right).$$ (12)

**Table 4:** Floc population characteristics for the unmixed (left, white) and mixed (right, grey) aggregation tank conditions.

	Unmixed tank conditions						
Test date (2017)	*M*_*ag*_ (%)	*M*_*ag*_ (%)	*FV*_*ag*_/*SV*_*ag*_	*FV*_*ag*_/*SV*_*ag*_	*ρ*_*a*_ (kg m^-3^)	*ρ*_*a*_ (kg m^-3^)	Test date (2017)
Feb. 17^th^	15.7±2.2	21.6±1.6	49.0±6.9	26.9±1.9	1035±145	1059±76	Feb. 22^nd^
Feb. 23^rd^	14.9±1.8	22.1±2.1	20.5±2.5	14.9±1.4	1078±131	1103±103	Feb. 28^th^
Mar. 2^nd^	17.7±1.9	26.0±2.0	14.8±1.6	11.1±0.8	1104±119	1137±83	Mar. 7^th^
Apr. 18^th^	17.8±3.4	21.0±3.7	14.7±2.5	8.0±1.4	1105±189	1188±202	Apr. 27^th^
				**Mixed tank conditions**			

The summations were performed over the LISST size bins excluding the first and last bins to avoid optical effects of out-of-range particles [46]. The percentage of particulate mass that aggregated, *M*_*ag*_, was calculated as *M*_*ag*_ = (*SV*_*ag*_/∑*V*_*pp*_)×*100*. Mixing the tank caused a larger percentage of the suspended particulate mass to aggregate into flocs ($\bar{M_{ag}}$ = 22.7% compared to the unmixed case with only $\bar{M_{ag}}$ = 16.5%). Despite this increase in aggregating mass, mixed conditions also consistently resulted in the creation of less relative floc volume (*FV*_*ag*_/*SV*_*ag*_, Table 4 columns 4 and 5). This comparison is summarized by defining a floc apparent density

$$\rho_{a}=\left(1-\varphi \right)\rho_{bent}+\varphi \rho_{water}.$$ (13)

Here, the porosity, *φ*, is calculated from the solid volume of aggregating particulate matter divided by the volume of newly formed flocs

$$\varphi =1-\frac{SV_{ag}}{FV_{ag}},$$ (14)

and the density of Bentonite was taken as *ρ*_*bent*_ = 2457 kg m^-3^. The apparent density for both tank conditions increased as the experiments progressed; however, the average floc apparent density was higher for the mixed conditions ($\bar{\rho_{a}}$ = 1122 kg m^-3^) compared to the unmixed conditions ($\bar{\rho_{a}}$ = 1081 kg m^-3^), where the values for each experiment and their uncertainties (calculated following [47]) are summarized in Table 4. A two-sample t-test was performed to quantify the significance of differences in these mean parameters calculated for each tank condition; resulting in p-values of 1.9×10^−14^ for the difference in average aggregated mass percentage ($\bar{M_{ag}}$), 3.6×10^−15^ for the difference in average relative floc volume ($\bar{{FV}_{ag}/{SV}_{ag}}$), and 0.32 for the difference in floc apparent density ($\bar{\rho_{a}}$). These results indicate statistically significant differences in the floc populations formed with the two tank conditions; though, the small difference in apparent density coupled with the larger uncertainties in this parameter indicate that there is a 68% chance that the average apparent densities are indeed different. While larger statistical samples of floc apparent density with lower uncertainty are desired, the results shown in Table 4 indicate that mixing the aggregation tank created flocs that were smaller and denser compared to the flocs formed in quiescent conditions.

**Fig 8:**
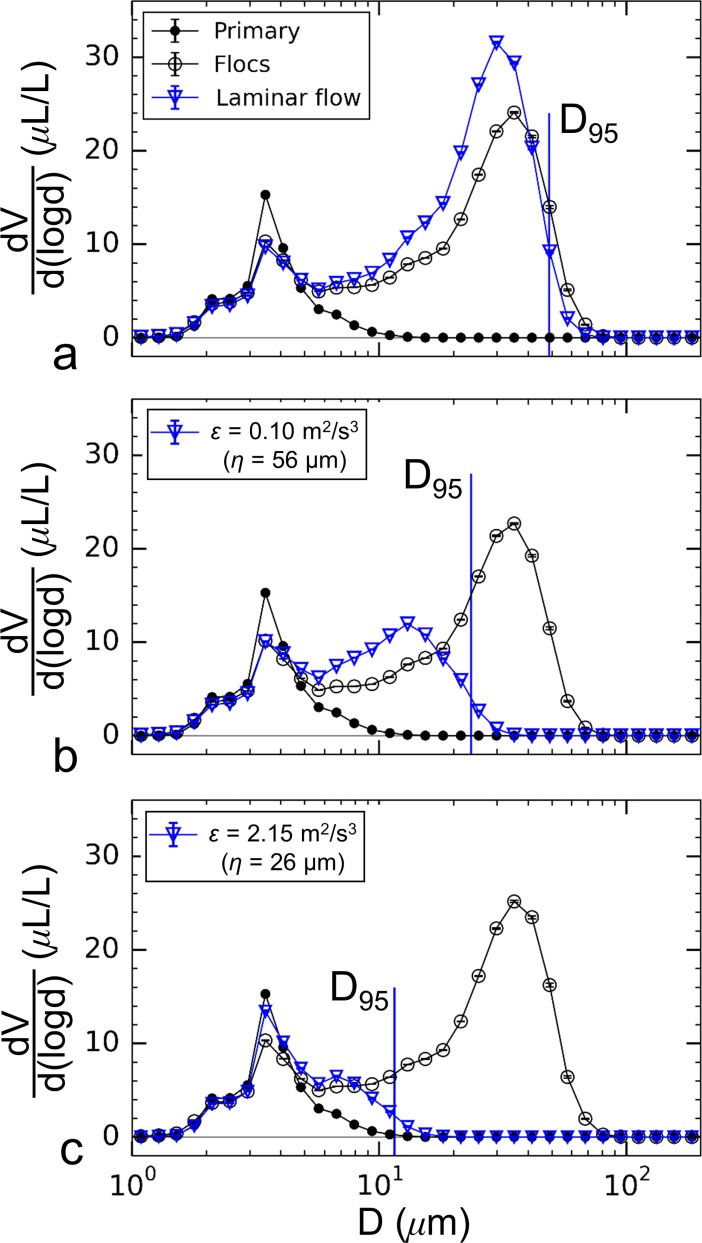
Size spectra of particle volume for the unmixed aggregation tank conditions. Data is plotted for (a) Laminar flow, (b) turbulent flow with *ε* = 0.10 m^2^/s^3^, and (c) turbulent flow with *ε* = 2.15 m^2^/s^3^ conditions. Spectra for the primary particles, flocs in the aggregation tank, and flocs in the breakup tank are plotted in each case.

The difference in floc populations summarized above resulted in distinct breakup responses due to turbulence. Particle volume spectra for the unmixed aggregation tank are shown in Fig 8 (Feb. 23^rd^, 2017), which plots spectra for the primary particles, flocs in the aggregation tank, and flocs in the breakup tank for three siphon flow conditions. Laminar flow through the siphon tubes resulted in an increase in floc volume, but the diameter corresponding to the peak volume decreased from 35 μm in the aggregation tank to 29.8 μm after flowing through the siphons. There was little change in the overall range of diameters of the floc population (Fig 8A). The four largest measurement bins where flocs were detected (*i*.*e*. *D* = 41.5, 49.0, 57.8, and 68.2 μm) decreased in volume. As a result, the largest stable floc size, defined here as *D*_*95*_, decreased slightly from 55.3 to 48.7 μm. This trend may indicate that a small amount of breakup occurred due to laminar shear experienced at the tube entrance or along its flow length. Thus, the observed increase in particle volume at intermediate sizes was likely the result of fracture of the largest flocs as they passed through the siphons, even though shear was quite small. The overall volume-weighted average floc size increased slightly from 16.6 μm to 16.8 μm due to laminar flow through the siphon tubes.

Turbulent flow with a dissipation rate of *ε* = 0.10 m^2^/s^3^ (Fig 8B) caused a large shift in the second peak in particle volume concentration to smaller diameters with a peak in volume concentration occurring at 13.0 μm for this flow condition. The largest stable floc diameter decreased to *D*_*95*_ = 23.5 μm. Increasing the dissipation rate to 2.15 m^2^/s^3^ (Fig 8C) resulted in a further decrease in size. In this case, *D*_*95*_ decreased to 11.5 μm and the spectrum nearly reverted back to the primary particle distribution. For these two turbulent flow cases, the volume-weighted average floc size decreased from 16.6 μm within the aggregation tank to 7.8 μm and 4.6 μm for particles subjected to higher turbulence dissipation rates of *ε* = 0.10 m^2^/s^3^ and 2.15 m^2^/s^3^, respectively.

**Fig 9:**
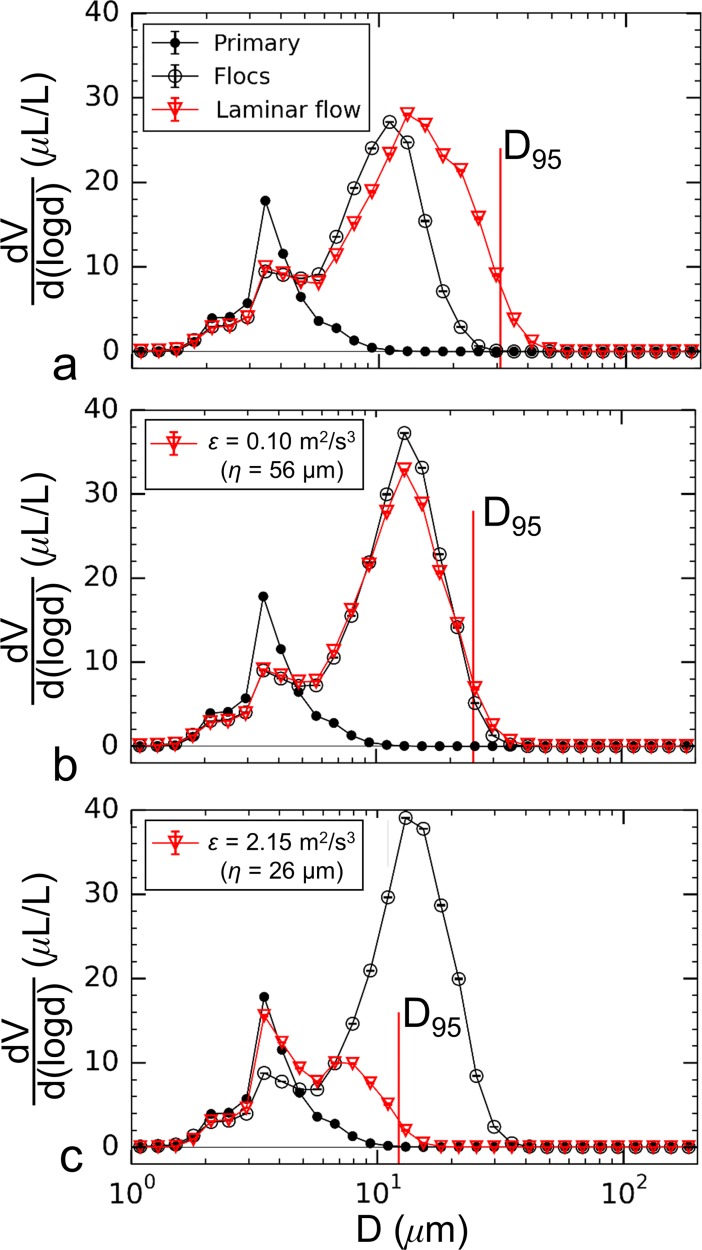
Size spectra of particle volume for the mixed aggregation tank conditions. Data is plotted for (a) laminar flow, (b) turbulent flow with *ε* = 0.10 m^2^/s^3^, and (c) turbulent flow with *ε* = 2.15 m^2^/s^3^ conditions. Spectra for the primary particles, flocs in the aggregation tank, and flocs in the breakup tank are plotted in each case.

Mixing within the aggregation tank using the impeller created smaller flocs compared to the unmixed aggregation tank, where the volume-weighted average floc size was 8.2 μm prior to flow through the siphon tubes (Fig 9A, Mar. 7^th^, 2017), compared to 16.6 μm for the unmixed conditions (Fig 8). In contrast to the unmixed condition, laminar flow resulted in an increase in the largest floc sizes; the volume-weighted average floc size increased from 8.2 μm to 11.2 μm and the maximum stable floc size increased from *D*_*95*_ = 18.9 μm in the aggregation tank to 30.9 μm after the siphon flow. Increased floc sizes indicate additional aggregation within the siphons and a lack of floc breakup. Turbulent flow with a dissipation rate of *ε* = 0.10 m^2^/s^3^ also did not cause reductions in floc sizes for the mixed tank conditions (Fig 9B). The volume-weighted average floc size remained unchanged at 10.2 μm before and after flowing through the siphons for this case with *D*_*95*_ increasing from 23.8 μm to 24.9 μm. Only with higher dissipation rates did a reduction in size occur, as shown in Fig 9C for *ε* = 2.15 m^2^/s^3^. For this case presented, decreases in volumetrically-weighted average and maximum floc sizes were from 10.9 μm to 5.2 μm and 25.0 μm to 12.2 μm, respectively.

**Fig 10:**
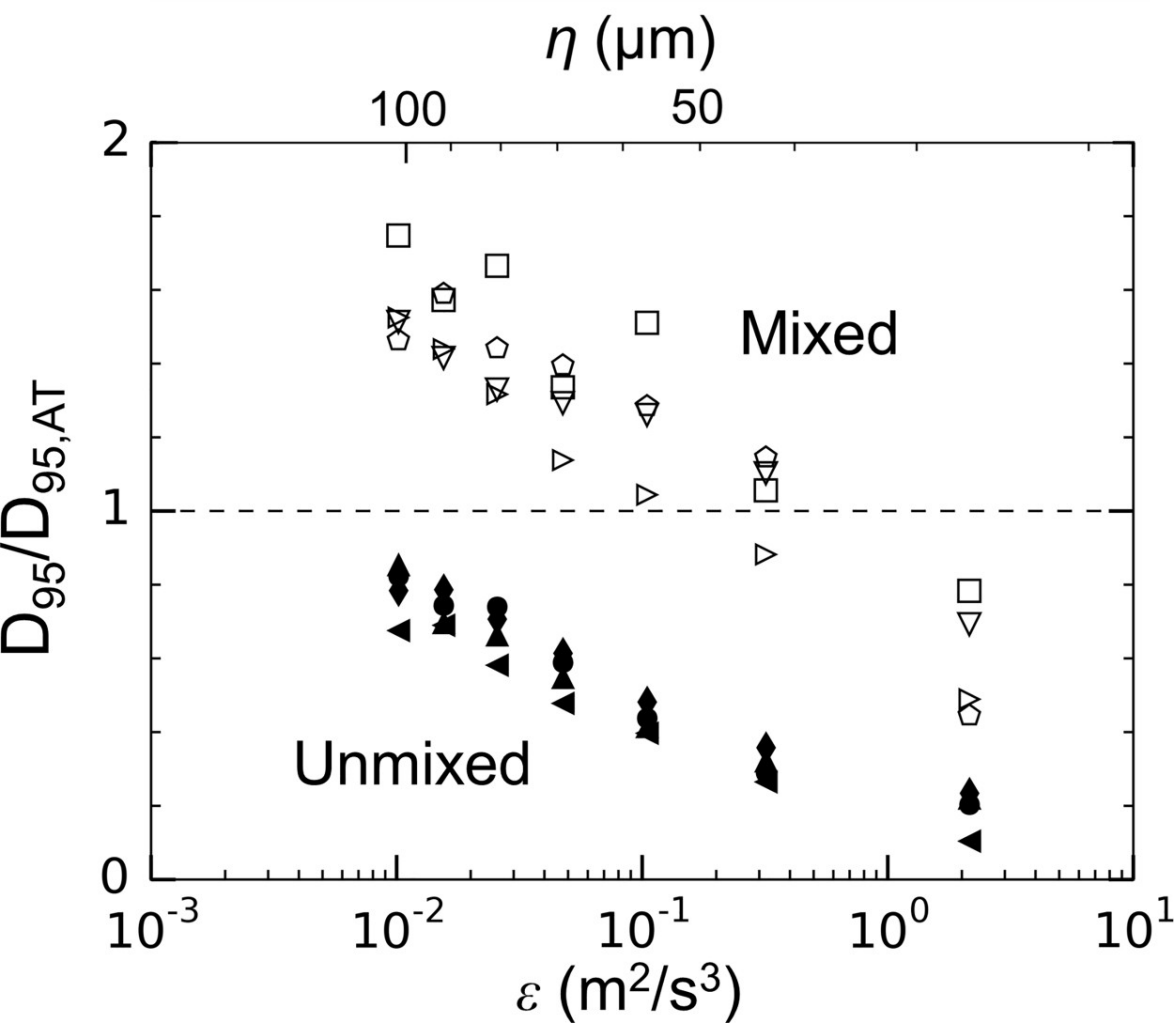
Change in largest floc size with turbulent dissipation rate in the siphon tubes. Symbols are consistent with Table 3.

The largest floc size measured after flow through the siphon tubes for each flow condition normalized with floc size measured in the aggregation tank (*D*_*95*,*AT*_) are plotted against turbulence dissipation rate in the siphon tubes (Fig 10). Two distinct regimes are apparent. The unmixed conditions resulted in a decrease in floc size at all dissipation rates and mixed conditions resulted in increases in floc size at low dissipation rates. In both cases, the trends were repeatable.

**Fig 11:**
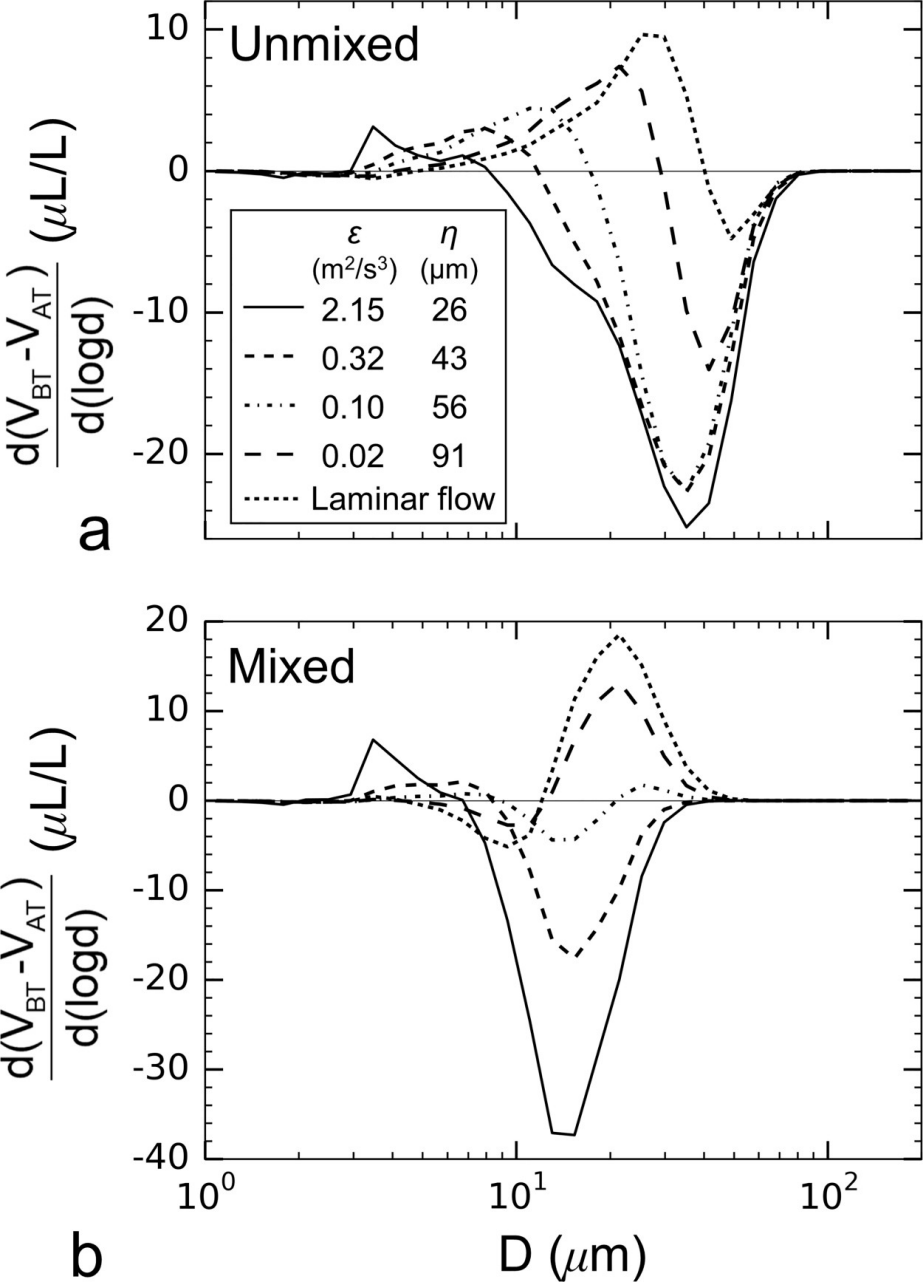
The difference in particle volume after flowing through the siphon tubes. Data for (a) an unmixed aggregation tank condition and (b) a mixed aggregation tank condition.

Generally, turbulence promotes aggregation up to the point that shear-induced breakage occurs. The results suggest that mixing within the aggregation tank may have caused floc breakup prior to flow through the siphons. Laminar flow and turbulence with a low dissipation rate within the siphons could then promoted additional floc formation. This would explain why mixed conditions resulted in less total floc volume (and higher floc apparent densities) even though more primary particle volume was aggregating (Table 4). This interpretation is also supported by the difference in particle volume spectra between the breakup and aggregation tanks (Fig 11). For the unmixed case (Fig 11A), the redistribution of particle mass showed a decrease in volume at larger floc sizes and an increase in particle volume at smaller floc sizes. In contrast, the mixed case did not initially follow this same trend. For laminar flow and a dissipation rate of *ε* = 0.02 m^2^ s^-3^, floc mass decreased at smaller floc sizes and increased at larger floc sizes, indicating aggregation. This growth regime persisted until breakup began to govern the dynamics at a siphon dissipation rate of *ε* = 0.32 m^2^ s^-3^ (Fig 10).

**Fig 12:**
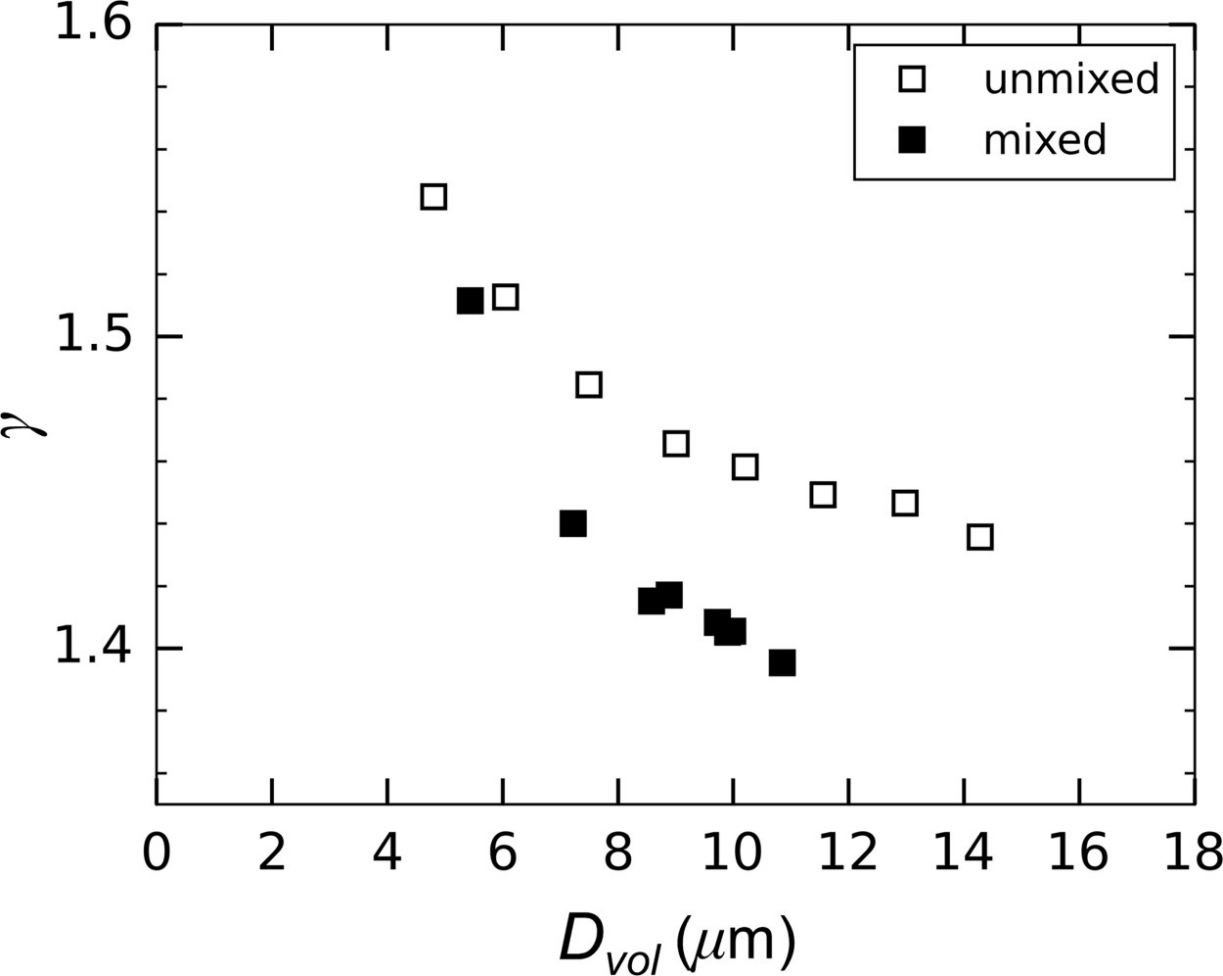
Response of the spectral slope of attenuation with changes in the particle volume-weighted mean diameter after flowing through the siphon tubes.

A comparison of the spectral slope of attenuation for both the unmixed and mixed aggregation tank conditions is plotted in Fig 12 versus particle diameter, *D*_*vol*_. In both cases, a consistent increase in the slope of spectral attenuation was measured concurrently with the decrease in floc size, plotted here as the volume-weighted mean diameter of the particle population obtained from the LISST. This result supports the hypotheses arising from the field data [13,14] that meaningful changes in floc size are detectable in the particle attenuation signature. However, the fact that the data for the unmixed and mixed case do not fall on the same curve suggests that the spectral slope may depend on subtler variations of the particle population than just the mean diameter, possibly in relation to floc shape, internal structure, and how the material is distributed as a function of size.

### Floc breakup strength characterization

**Fig 13:**
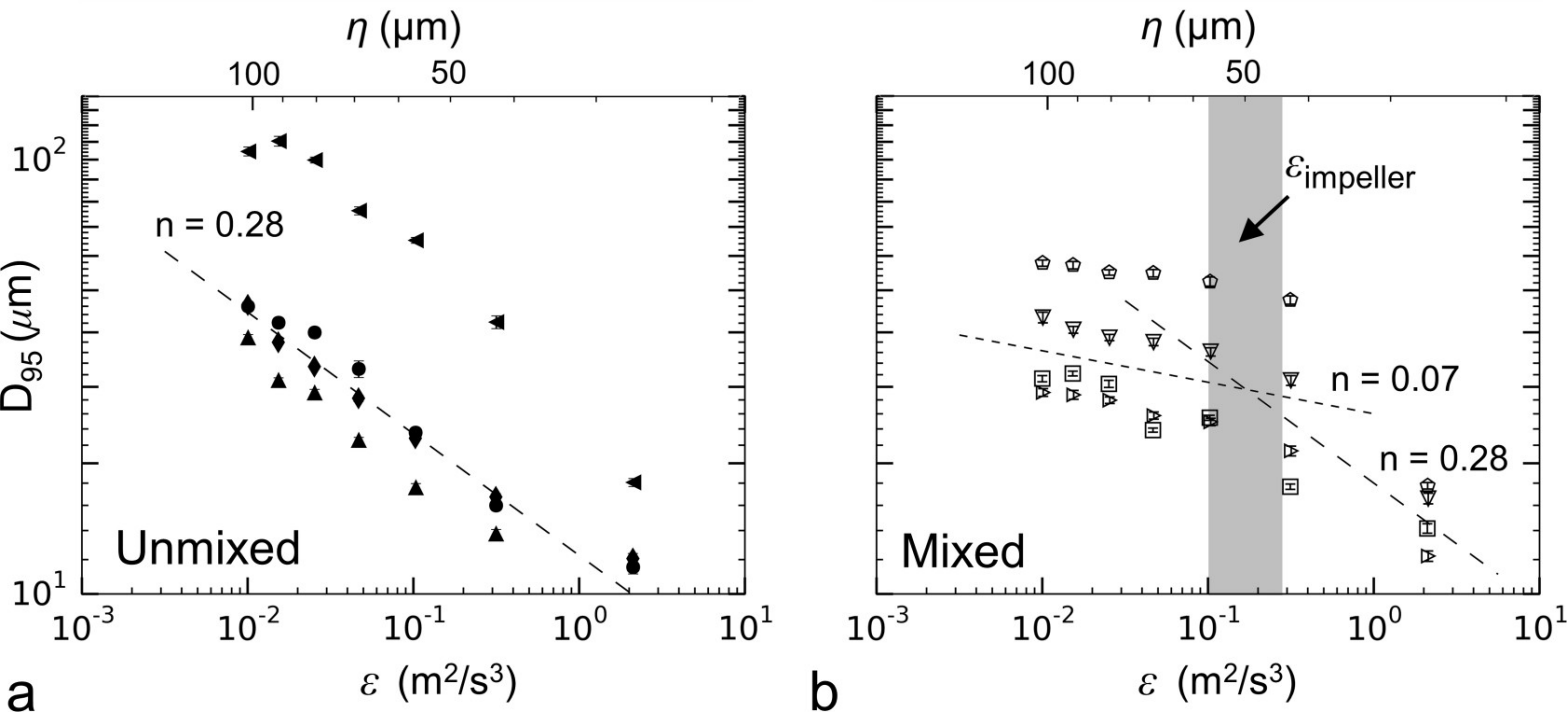
Plots of largest stable floc size vs. dissipation rate in the siphon tubes. (a) Unmixed aggregation tank conditions and (b) the mixed aggregation tank conditions are shown. Symbols consistent with Table 3.

Data used to obtain strength exponents following Eq (6) for the unmixed aggregation tank conditions showed a large spread in *D*_*95*_ across experimental runs (Fig 13A). This trend indicates a large spread in the floc strength coefficient, *C*. It has been hypothesized that the strength coefficient is representative of the overall floc strength [48], meaning that the flocs became progressively weaker with time as the experiments progressed. Despite this spread, the data showed very repeatable strength exponents, with *n* = 0.28 plotted for comparison. Fits to the data indicated an average strength exponent of *n* = 0.28 ± 0.07, with the fit to each data run given in Table 3. These results are similar to the value of *n* = 0.25 predicted theoretically [27] and observed in past experimental studies (Table 1), though the current data showed more repeatable strength exponents than has been observed in previous experiments with clay flocs.

What has not yet been widely observed in the literature is the response for the flocs grown in the mixed aggregation tank (Fig 13B), which displays a non-constant strength exponent. At dissipation rates below 0.1 m^2^/s^3^, the maximum floc size decreased with a reduced strength exponent of *n* = 0.07 ± 0.06. Above dissipation rates of 0.1 m^2^/s^3^, the breakup response appeared to revert back to that observed for the unmixed case with the data more closely following the slope of *n* ≈ 0.27; however, additional data points at dissipation rates above 0.3 m^2^/s^3^ are needed to confirm this trend. Francois [35] observed a breakup response for kaolinite particles flocculated with aluminum sulfate that also exhibited two distinct regions with different strength exponents; however, their strength exponents were substantially higher than the current results (*n* = 0.15–0.25 compared to *n* = 0.08 over a similar range in dissipation rate). Considering that floc size increased during these siphon flow conditions (Fig 10), the low strength exponent is likely representative of the floc growth regime rather than actual breakup.

As a means of explaining the observed change in floc breakup response further, the dissipation rate caused by the turbulence of the mixing impeller was estimated using the following expression [30]

$$\varepsilon =\frac{P_{o}R_{i}{}^{3}D_{i}{}^{5}}{V_{sw}},$$ (15)

where the total impeller power number (*P*_*o*_) is estimated following [49], *R*_*i*_ is the impeller rotation speed, *D*_*i*_ is the impeller diameter, and *V*_*sw*_ is the total swept volume of the mixing impeller. The dissipation rate of the impeller was calculated to be 0.22 ± 0.08 m^2^/s^3^, which is an estimate of the maximum dissipation experienced by the flocs while residing in the aggregation tank under mixed conditions. This impeller dissipation rate is shown in Fig 13B as a shaded area. Even considering the large amount of uncertainty in this estimate, the dissipation of the impeller fell very close to the values where the change in breakup behavior was observed. Mixer-induced turbulence, thus, effectively defined the upper limit for floc strength. For significant breakage to occur, the flocs had to be subjected to dissipation rates greater than what was experienced during their formation. Notably, this non-constant strength exponent trend and the dissipation rate at which breakup became significant was consistent across experimental runs, with a marked reduction in floc size observed after the data taken with *ε* = 0.32 m^2^/s^3^. This repeatability occurred despite the large differences in floc sizes; data taken on Feb. 22^nd^, 2017 show largest floc sizes of *D*_*95*_ = 47.5 μm while data taken on Apr. 27^th^, 2017 show largest floc sizes of only *D*_*95*_ = 17.7 μm at this value of dissipation rate.

## Discussion

### Evolution of floc size during experimentation

Aging is one possible explanation for the flocs weakening with time. Aging has been studied by Francois [50], but the cementation process reported resulted in smaller flocs with increased strength, in contrast to the weakened flocs observed here. It has also been shown that increasing flocculant concentration can increase the floc strength coefficient [51]. In the current experiment, the concentration of flocculant (NaCl) increased over the course of the experimental period from *S* = 10.04 psu measured on Feb. 17^th^ 2017 to *S* = 11.06 psu on April 27^th^ 2017. For salinity to be the cause of the decrease in strength coefficient observed presently, floc strength would have to be inversely proportional to flocculant concentration. The possibility of the effect of surfactants or other organic contaminants acting to decrease surface activity of the bentonite particles with time cannot be ruled out, although no direct evidence of this was observed. In the natural ocean environment, the effects of organics or other surfactants could lead to a large range in floc strength coefficients for the same type of primary aggregating particles [52]. Interestingly, the cause of the change in strength coefficient apparently has little to no effect on the strength exponent and floc breakup mechanism, suggesting that the exponent *n* will provide the most utility for future marine breakup models.

### Particle lengthscales relative to turbulent eddies

**Fig 14:**
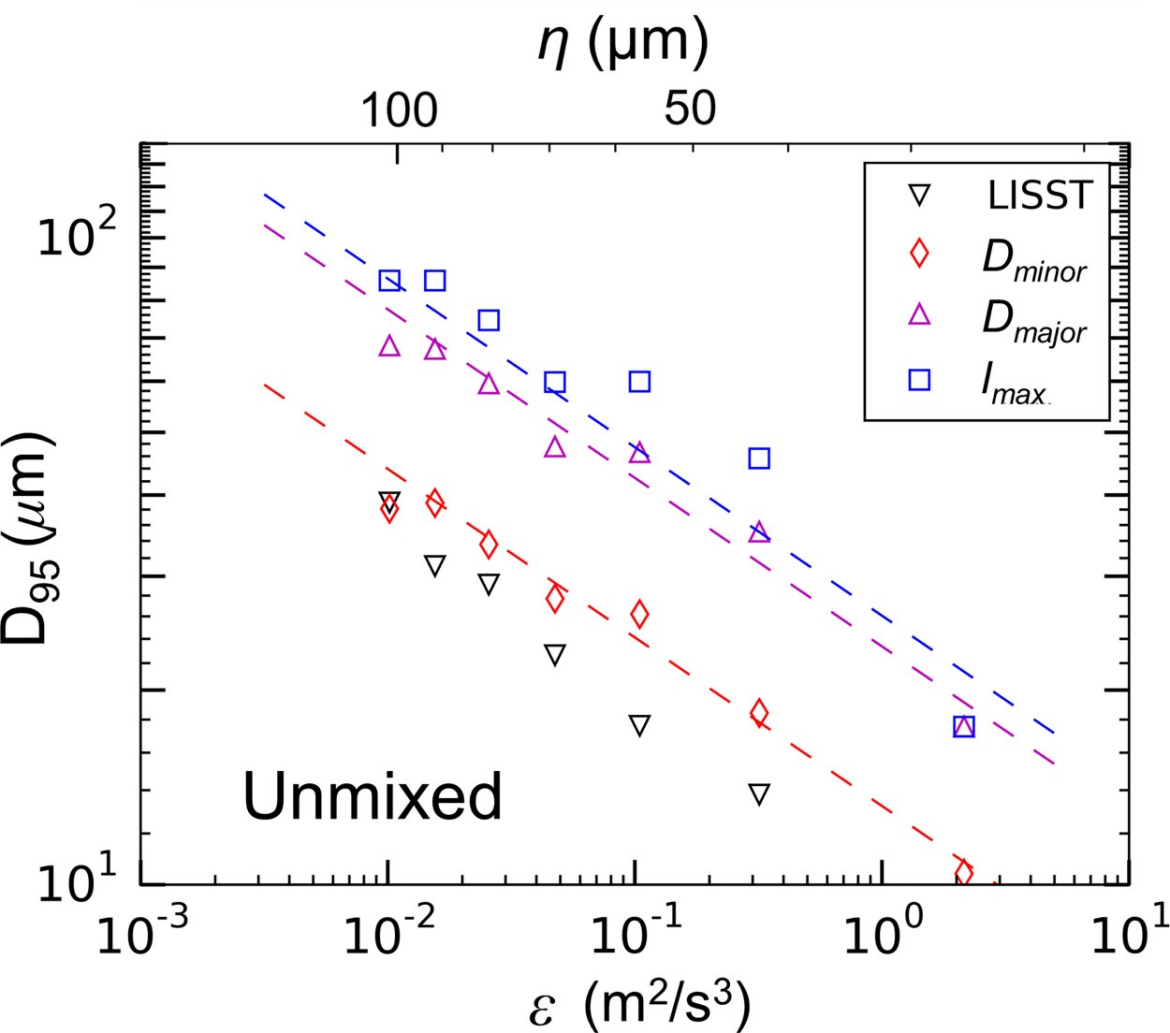
Floc strength curves plotted with lengthscales obtained from the LISST and from the image analysis for an unmixed aggregation tank.

To investigate if strength curves obtained from the largest floc dimensions, rather than those output by the LISST, affected the breakup trends, representative strength curves were obtained using microscope data. *D*_*95*_ was calculated from the minor-axis (*D*_*minor*_), major-axis (*D*_*major*_), and Feret-diameter (*l*_*max*_) spectra (see Fig 5 as an example) for the different siphon flow conditions (Fig 14). The overall breakup trends were preserved regardless of the lengthscale used for the analysis; strength exponents were constant with values of *n* = 0.26 for the minor-axis spectra, *n* = 0.26 for the major-axis spectra, and *n* = 0.30 for the Feret-diameter spectra. This result is consistent with [32], who have shown that similar strength exponents are determined from using the volume-weighted floc diameter instead of the maximum stable floc diameter. Thus, while the LISST observations miss the largest lengthscales of the flocs, the data can still be used to quantify floc breakup.

**Fig 15:**
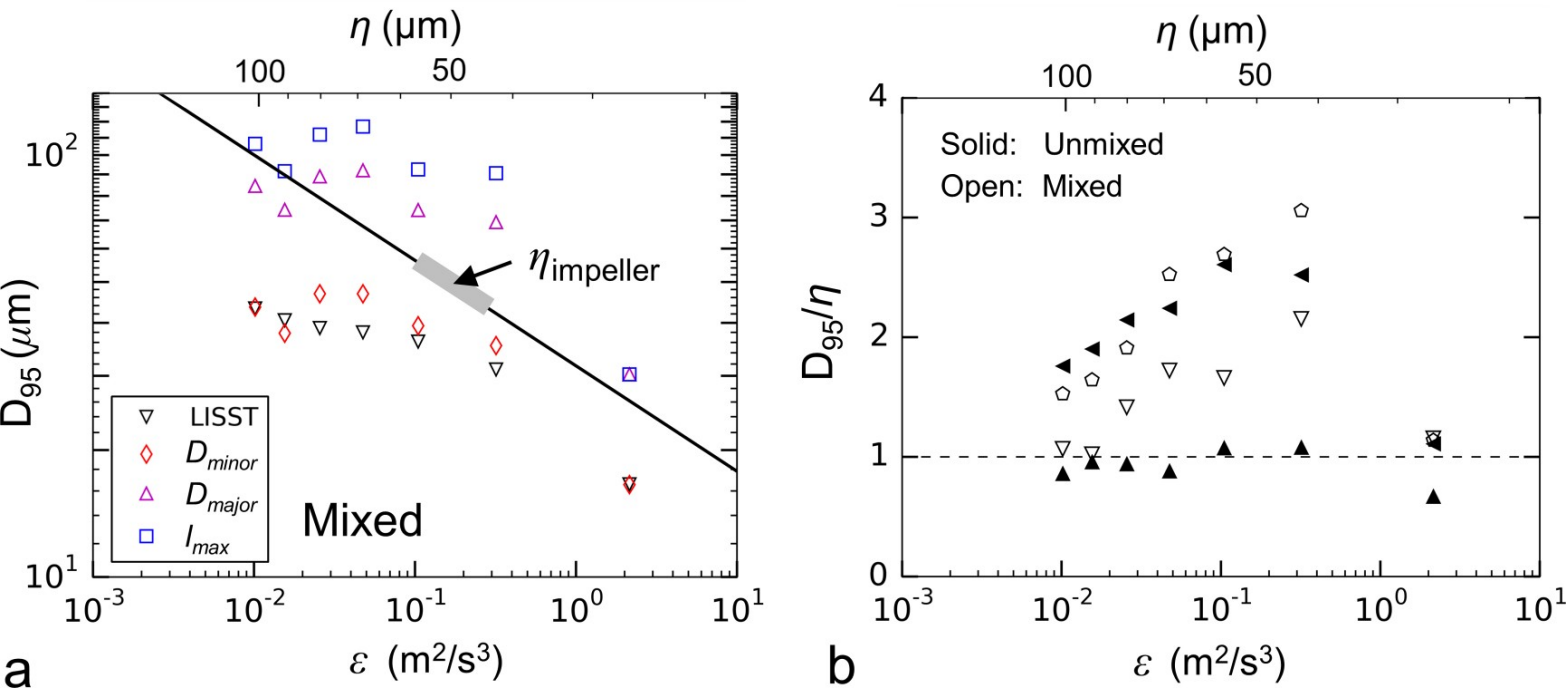
Effects of particle lengthscale on the breakup trends. (a) Floc strength curves plotted with lengthscales obtained from the LISST and from the image analysis for a mixed aggregation tank condition. Kolmogorov lengthscale of the siphon flow plotted as the solid black line. (b) *D*_*95*_ of the Feret-diameter particle spectra normalized with the Kolmogorov lengthscale of the siphon flow and plotted against the dissipation rate in the siphon tubes. Symbols in (b) consistent with Table 3.

It has been suggested previously that the Kolmogorov length provides a size limit or scaling parameter for some flocculating materials [53,54]. One hypothesis to explain the non-constant strength exponent (Fig 13B) is that strong flocs continue to aggregate until they reach this Kolmogorov lengthscale, at which point breakage occurs. Breakup trends for the mixed aggregation tank using representative LISST and microscope measurements (Fig 15A) showed how the maximum floc lengthscale (*l*_*max*_) exceeded the Kolmogorov length for many of the flow conditions. Comparing data across experiments, there also did not appear to be a consistent correlation between maximum floc size and eddy size for either the unmixed or mixed aggregation tank conditions (Fig 15B). As the flocs weakened with time (as described in the preceding section), they reduced in size until their maximum length was approximately the same as the Kolmogorov length. Given the observed temporal variability, the results do not point to a universal relationship and additional decreases in floc size below the Kolmogorov length cannot be discounted. Similarly, Cross *et al*. [55] compared the size of flocs of suspended particulate material in the Western English Channel to the Kolmogorov lengthscale in the surrounding waters and found that while the natural flocs were typically smaller than the Kolmogorov length, eddy size did not serve as an absolute limit to floc size.

### Particle structure

The non-constant strength exponent of the flocs grown in the aggregation tank under different mixing conditions (Fig 13) suggests that mixing prior to siphon flow causes a fundamental alteration of the strength that should be displayed in the floc structure. Mixing was shown to increase the floc apparent density (Table 4); a corresponding small increase in the fractal dimension of the flocs would be expected since floc density is a function of fractal dimension according to $\rho \propto l_{max}^{D_{f3}‑3}$ [26]. The microscope images obtained allow quantification of the floc structure by measuring floc perimeters, lengthscales, and projected areas. These parameters can be correlated to the fractal dimension of the floc [56]. Because the out-of-plane dimension of the flocs was not viewed, only the two-dimensional fractal dimension (*D*_*f2*_) was investigated. *D*_*f2*_ typically correlates positively to the three-dimensional fractal dimension [57] such that it is still a valid parameter from which to compare overall floc structure. *D*_*f2*_ was calculated as the slope of the floc projected area, *A*_*f*_, plotted with respect to the maximum floc length on a log-log slope, as given by [58]

$$A_{f}\propto l_{\max}^{D_{f2}}.$$ (16)

Values for *D*_*f2*_ can theoretically range between 1.0 and 2.0, with values closer to 1.0 indicating a more-fractal floc. For the unmixed condition, *D*_*f2*_ = 1.45 ± 0.29, while mixing resulted in *D*_*f2*_ = 1.56 ± 0.30. While this difference in *D*_*f2*_ may seem small given the markedly different breakup response observed, the difference (Δ*D*_*f2*_ = 0.11) is similar to previous reports of clay flocculation as a function of turbulent energy. Logan and Kilps [58] observed an increase in fractal dimension from *D*_*f2*_ = 1.68 to 1.89 (Δ*D*_*f2*_ = 0.21) for carboxylate microspheres flocculated in a rolling cylinder and paddle-mixed tank, respectively. They attributed the higher fractal dimension to floc restructuring from the higher shear rate near the paddle mixer. Spicer *et al*. [59] formed flocs in a paddle-mixed jar with varying rates of shear. After mixing with a high-shear rate, *G* = 300 s^-1^, they measured a fractal dimension of *D*_*f3*_ = 2.65, compared to 2.4 for flocs created with a low shear rate, *G* = 50 s^-1^ (Δ*D*_*f3*_ = 0.25). Given the effect turbulence has on floc structure and the apparent strong effect that floc structure has on breakup strength, an improved understanding of how turbulence influences floc structure during growth will be critical in the ultimate formulation of floc breakup models.

### Implications to marine particle dynamics

Natural marine aggregates span a large range in size, from less than O^-6^ m to more than O^-2^ m, and can be composed of both organic (living and dead) and inorganic matter [19]. Formation mechanisms can be primarily through electrostatic forces as in the case of clay particles [60], or more often can include biological factors such as exudates that serve as organic glues to increase the probability and strength of particle cohesion [61]. Thus, our laboratory observations of uniform clay particles exposed to a comparatively high range in turbulence (0.01< *ε* < 2 m^2^ s^-3^) represent a small portion of the aggregate formation and disruption scenarios found in nature. In this case, how might our observations inform the nature of particle interactions within marine systems?

There are two conceptual models for aggregate disruption due to exposure to hydrodynamic shear force; the erosion of individual, primary component particles from the surface of the floc and floc fracture into two or more smaller aggregates [62]. If erosion dominates, floc mass (and associated volume) will decrease and the eroded mass will be added to the size range of the primary component particles. Conversely, if fracture dominates, sub-aggregates will increase the particulate mass for intermediate particle sizes between the disrupting floc and primary particles. While both breakup processes would be expected to decrease sinking rate, erosion would be expected to have a larger impact since more mass would be shifted to slower sinking primary particles. The laboratory results indicate that both break-up processes occurred simultaneously, but that fracture dominated at low dissipation rates. For the unmixed aggregation tank conditions (Fig 11A), little primary particle volume was created at the lowest dissipation rates (*ε* = 0.02 to 0.10 m^2^/s^3^), but larger increases in primary particle volume were observed for dissipation rates of *ε* = 0.32 and 2.15 m^2^/s^3^. Similarly, for flocs formed under active stirring, disruption was initially delayed until higher turbulent energy was applied, but once disruption started, the redistribution of particulate mass was generally the same; fracture when exposed to relatively low turbulent energy and erosion when exposed to higher energy (Fig 11B). Such a sequence in marine floc disruption versus turbulence would tend to dampen the effect of break-up on sinking rate under low turbulence conditions and enhance the impact under more energetic conditions.

As was previously reported [24], floc strength and apparent density are both impacted by the level of turbulence during formation. More energetic collisions between coalescing particles appear to result in more compaction between aggregated particles and greater cohesion. Active mixing within the aggregation tank resulted in flocs of smaller average size, greater strength, larger fractal dimension, and greater floc apparent density. If this is true for marine particles, then the impact on vertical particulate carbon flux is perhaps more significant than the size redistribution of particulate mass resulting from disruption. For a given particulate mass concentration, flocs formed under more sustained energetic conditions (but, not so energetic as to cause disruption to dominate) should be expected to sink faster than those formed under more quiescent conditions and be less likely to disrupt when exposed to higher turbulent energy, and therefore lose less mass to respiration and remineralization during the sinking process. Thus, excluding episodic high-energy storm events, regions of the global ocean exposed to higher average wind stress and, therefore, greater average near-surface turbulence should produce flocs that sink more rapidly into the ocean interior than more quiescent regions.

Sinking rates of marine snow are poorly correlated with the physical dimensions of flocs [63], suggesting that poor knowledge of the apparent density of natural flocs is a large source of uncertainty in modeling the vertical flux of particulate matter. Biological processes are one factor influencing floc apparent density. Eppley *et al*. [64] conducted laboratory experiments that illustrated how phytoplankton senescence at the time of floc formation impacted the apparent density and sinking rate of the resulting flocs. Dead cells resulted in denser and more rapidly sinking flocs compared with live cells. Microbial consumption during sinking can also alter floc structure and apparent density as organic components are gradually remineralized [65]. In addition to these biological controls, our data suggests that turbulence at the time of aggregate formation is also of importance. If the level of turbulent energy affects aggregate structure and, consequentially, floc apparent density and strength, then knowledge of the turbulence history of the various floc components may help to more-fully explain variations in sinking rate and lead to more realistic projections of particulate matter transport to the ocean interior.

## Conclusion

Flocs of bentonite clay particles in salt water were grown in the laboratory and their breakup response to turbulent pipe flow was quantified. Flocs were grown with unmixed and mixed aggregation conditions and the effect of these growth hydrodynamics on the resulting particle strength was quantified. Floc breakup was characterized with forward particle scattering function obtained from a LISST-100X, the spectral slope of particle attenuation obtained using ac-9 sensors, and from microscope images.

The LISST instrument was found to be insensitive to the largest lengthscales of the flocs in the flow; however, the effect of lengthscale used in the breakup analysis was not found to greatly influence the resulting strength exponent. Signatures of floc breakup were also detected in the spectral slope of attenuation, with spectral slope increasing with decreasing floc size.

The size of the smallest eddies in the flow, represented with the Kolmogorov lengthscale, did not appear to control the resulting largest floc length in the current investigation. Flocs both larger and smaller than the Kolmogorov length displayed repeatable breakup response with strength exponents ranging from *n* = 0.26 to 0.33.

The hydrodynamic conditions responsible for floc growth were found to have a large influence on floc strength. Flocs grown under mixed conditions displayed little to no breakup prior to being subjected to turbulence with a dissipation rate greater than that experienced during formation. The increased strength of these flocs was determined to be a result of an increase in the floc fractal dimension and floc apparent density, suggesting that turbulence during floc formation resulted in stronger, more compact flocs. Additional measurements of floc strength as well as structure from *in-situ* measurements in the field are identified as a need for the subsequent improvement of marine floc breakup modeling efforts.

## Abbreviations

A: aggregation terms in Eq (2)
A_f_: projected area of a floc image (m^2^)
B: breakup terms in Eq (2)
C: attenuation (m^-1^)
C: floc strength coefficient
c_p_: attenuation due to particulate mass (m^-1^)
$c_{p}^{*}$: mass-specific attenuation coefficient (m^2^ g^-1^)
${\bar{c}}_{p,LISST}$: average *c*_*p*_ measured by the LISST (m^-1^)
Δ*c*_*p*,*LISST*_: change in *c*_*p*_ measured by the LISST over the course of the experiment (%)
D: particle diameter (m)
D: siphon inside diameter (m)
D_95_: 95^th^ percentile diameter of the particle population (m)
${\bar{D}}_{95,LISST}$: average *D*_*95*_ measured with the LISST (m)
ΔD_95,LISST_: Change in *D*_*95*_ over the course of the experiment (%)
D_i_: mixing impeller diameter (m)
D_f2_: two-dimensional fractal dimension
D_f3_: three-dimensional fractal dimension
D_max_: maximum stable diameter of the floc population (m)
D_major_: major-axis length of the ellipse fitted to a floc image (m)
D_minor_: minor-axis length of the ellipse fitted to a floc image (m)
D_vol_: volumetrically-weighted mean particle diameter (m)
FV_ag_: floc volume that is formed due to aggregation (μL L^-1^)
H: tank water level height (m)
G: shear rate (s^-1^)
K: breakup kernel
L: siphon length (m)
l_max_: maximum Feret diameter of the floc (m)
m: particle mass
M_ag_: percentage of primary particle mass that aggregates into flocs
N: floc strength exponent
N: particle number concentration
P_0_: impeller power number
Re: Reynolds number in the siphon tube (*Re* = *ud*/*ν*)
R_i_: impeller rotation speed (rpm)
S: salinity (psu)
S_io_: source term in Eq (2)
SV_ag_: solid particle volume that aggregates into flocs (μL L^-1^)
U: water velocity inside the siphon tubes (m s^-1^)
V: particle volume (μL)
V_f_: encased floc volume (μL)
V_sw_: swept volume of the mixing impeller (m^3^)
X: particulate mass concentration (g m^-3^)
W: settling term in Eq (2)
Α: particle stickiness parameter
Β: aggregation kernel
β_f_: near-forward particle scattering function
Γ: particle mass re-distribution function
Γ: spectral-slope of attenuation
Ε: dissipation rate of kinetic energy (m^2^ s^-3^)
Η: Kolmogorov lengthscale (m)
Θ: correlation constant for particle attenuation as a function of wavelength
λ: wavelength (nm)
ν: kinematic viscosity (m^2^ s^-1^)
ξ: slope of the particle number distribution (*ξ* = *γ* + 3)
τ_w_: wall shear stress (N m^-2^)
φ: floc porosity
ρ_a_: Apparent particle density (kg m^-3^)
AT: parameter in the aggregation tank
BT: parameter in the breakup tank
pp: parameter of the primary particles
LISST: parameter obtained from the LISST-100X measurements

## References

[1] Hsu JP, Glasgow LA. Floc size reduction in the turbulent environment. Part Sci Technol. 1983;1(2):205–22.

[2] Smith SJ, Friedrichs CT. Size and settling velocities of cohesive flocs and suspended sediment aggregates in a trailing suction hopper dredge plume. Cont Shelf Res. 2011;31:50–63.

[3] Yang MY, Chan JGY, Chan HK. Pulmonary drug delivery by powder aerosols. J Control Release. 2014;193:228–40. 10.1016/j.jconrel.2014.04.055 24818765 10.1016/j.jconrel.2014.04.055

[4] Fowler SW, Knauer GA. Role of large particles in the transport of elements and organic-compounds through the oceanic water column. Prog Oceanogr. 1986;16(3):147–94.

[5] Sarmiento JL, Hughes TM, Stouffer RJ, Manabe S. Simulated response of the ocean carbon cycle to anthropogenic climate warming. Nature. 1998;393:245–9.

[6] Logan BE. Settling velocities of fractal aggregates. Environ Sci Technol. 1996;30(6):1911–8.

[7] Diehl P, Haardt H. Measurement of the spectral attenuation to support biological research in a “plankton tube” experiment. Oceanologia. 1980;3(229):89–96.

[8] McCave IN. Size spectra and aggregation os suspended particles in the deep ocean. Deep Sea Res. 1984;31(4):329–52.

[9] Hawley N. Settling Velocity Distribution of Natural Aggregates. 1982;87:9489–98.

[10] Alldredge AL, Passow U, Logan BE. The abundance and significance of a class of large, transparent organic particles in the ocean. Deep Res Part I. 1993;40(6):1131–40.

[11] Alldredge AL, Silver MW. Characteristics, dynamics and significance of marine snow. Prog Ocean. 1988;20:41–82.

[12] Agrawal YC, Pottsmith HC. Instruments for particle size and settling velocity observations in sediment transport. Mar Geol. 2000;168(1–4):89–114.

[13] Boss E, Pegau WS, Gardner WD, Zaneveld JR V., Barnard AH, Twardowski MS, et al Spectral particulate attenuation and particle size distribution in the bottom boundary layer of a continental shelf. J Geophys Res. 2001;106(C5):9509–16.

[14] Ackleson SG. Optical determinations of suspended sediment dynamics in western Long Island Sound and the Connecticut River plume. J Geophys Res. 2006;111(C7):C07009.

[15] Slade WH, Boss E, Russo C. Effects of particle aggregation and disaggregation on their inherent optical properties. Opt Express. 2011;19(9):7945–7959. 10.1364/OE.19.007945 21643044 10.1364/OE.19.007945

[16] Ramkrishna D. Population balances: Theory and applications to particulate systems in engineering. San Diego, CA: Academic Press; 2000.

[17] Dubovskii PB, Stewart IW. Existence, uniqueness and mass conservation for the coagulation-fragmentation equation. Math Methods Appl Sci. 1996;19:571–91.

[18] White WH. A global existence theorem for Smoluchowski’s coagulation equations. Proc Am Math Soc. 1980;80(2):273–6.

[19] Burd AB, Jackson GA. Particle aggregation. Ann Rev Mar Sci. 2009;1(1):65–90. 10.1146/annurev.marine.010908.163904 21141030

[20] Saffman PG, Turner JS. On the collision of drops in turbulent clouds. J Fluid Mech. 1956;1(1):16–30.

[21] Friedlander SK. Smoke, dust and haze: Fundamentals of aerosol behavior. New York: Wiley-Interscience; 1977.

[22] Ramkrishna D. The status of population balances. Rev Chem Eng. 1985;3(1):49–95.

[23] Flesch JC, Spicer PT, Pratsinis SE. Laminar and turbulent shear induced flocculation of fractal aggregates. AIChE J. 1999;45(5):1114–24.

[24] Jiang Q, Logan BE. Fractal dimensions of aggregates from shear devices. Journal-American Water Work Assoc. 1996;100–13.

[25] Logan BE, Wilkinson DB. Fractal geometry of marine biological aggregates. 1990;35(1):130–6.

[26] Jiang Q, Logan BE. Fractal dimensions of aggregates determined from steady state size distributions. Environ Sci Technol. 1991;25:2031.

[27] Parker DS, Kaufman WJ, Jenkins D. Floc breakup in turbulent flocculation processes. J Sanit Eng Div Proc Am Soc Civ Eng. 1972;98(1):79–99.

[28] Pandya JD, Spielman LA. Floc breakage in agitated suspensions: Theory and data processing strategy. J Colloid Interface Sci. 1982;90(2):517–31.

[29] Jeffrey DJ. Aggregation and break-up of clay flocs in turbulent flow. Adv Colloid Interface Sci. 1982;17:213–8.

[30] Spicer PT, Pratsinis SE. Coagulation and fragmentation: Universal steady-state particle-size distribution. AIChE J. 1996;42(6):1612–20.

[31] Kramer TA, Clark MM. Incorporation of aggregate breakup in the simulation of orthokinetic coagulation. J Colloid Interface Sci. 1999;216(1):116–26. 10.1006/jcis.1999.6305 10395769 10.1006/jcis.1999.6305

[32] Jarvis P, Jefferson B, Gregory J, Parsons SA. A review of floc strength and breakage. Water Res. 2005;39(14):3121–37. 10.1016/j.watres.2005.05.022 16000210 10.1016/j.watres.2005.05.022

[33] Hinze JO. Fundamentals of the hydrodynamics mechanisms of splitting in dispersion process. AIChE J. 1955;1(3):289–95.

[34] Alldredge AL, Granata TC, Gotschalk CC, Dickey TD. The physical strength of marine snow and its implications for particle disaggregation in the ocean. Limnol Oceanogr. 1990;35(7):1415–28.

[35] Francois RJ. Strength of aluminum hydroxide flocs. Water Res. 1987;21(9):1023–30.

[36] Tambo N, Hozumi H. Physical characteristics of flocs II: Strength of floc. Water Res. 1979;13(1970):421–7.

[37] Bouyer D, Line A, Cockx A, Do-Quang Z. Experimental analysis of floc size. Trans Inst Chem Eng. 2001;79:1017–24.

[38] Li T, Zhu Z, Wang D, Yao C, Tang H. The strength and fractal dimension characteristics of alum-kaolin flocs. Int J Miner Process. 2007;82(1):23–9.

[39] Zhu Z, Wang H, Yu J, Dou J. On the kaolinite floc size at the steady state of flocculation in a turbulent flow. PLoS One. 2016;11(2):1–16. 10.1371/journal.pone.0148895 PMC4763281 26901652

[40] Thorpe SA. The turbulent ocean. Cambridge University Press; 2005.

[41] Bakewell HP, Lumley JL. Viscous sublayer and adjacent wall region in turbulent pipe flow. Phys Fluids. 1967;10(9):1880–9.

[42] Schlichting H, Gersten K, Krause E, Oertel H, Mayes K. Boundary-layer theory. Vol. 7 Springer; 1955.

[43] Agrawal YC, Whitmire A, Mikkelsen OA, Pottsmith HC. Light scattering by random shaped particles and consequences on measuring suspended sediments by laser diffraction. J Geophys Res Ocean. 2008;113(4):1–11.

[44] Pegau S, Zaneveld JR V, Mitchell BG, Mueller JL, Kahru M, Wieland J, et al Ocean optics protocols for satellite ocean color sensor validation, revision 4, volume IV: Inherent optical properties: Instruments, characterizations, field measurements and data analysis protocols. NASA Tech Memo. 2003;211621.

[45] Schindelin J, Arganda-Carreras I, Frise E, Kaynig V, Longair M, Pietzsch T, et al Fiji: An open-source platform for biological-image analysis. Nat Methods. 2012;9(7):676–82. 10.1038/nmeth.2019 22743772 10.1038/nmeth.2019 PMC3855844

[46] Graham GW, Davies EJ, Nimmo-Smith WAM, Bowers DG, Braithwaite KM. Interpreting LISST-100X measurements of particles with complex shape using digital in-line holography. J Geophys Res Ocean. 2012;117(5):1–20.

[47] Bevington PR, Robinson KD. Data reduction and error analysis for the physical sciences. McGraw-Hill; 1992.

[48] Bache DH. Floc rupture and turbulence: A framework for analysis. Chem Eng Sci. 2004;59(12):2521–34.

[49] Paul EL, Atiemo-Obeng VA, Kresta SM. Handbook of industrial mixing: science and practice. John Wiley & Sons; 2004.

[50] Francois RJ. Ageing of aluminum hydroxide flocs. Water Res. 1987;21(5):523–31.

[51] Leentvaar J, Rebhun M. Strength of ferric hydroxide flocs. Water Res. 1983;17(8):895–902.

[52] Van Olphen H. An introduction to clay colloid chemistry: for clay technologists, geologists, and soil scientists. Wiley-Interscience; 1977.

[53] Fugate DC, Friedrichs CT. Controls on suspended aggregate size in partially mixed estuaries. Estuar Coast Shelf Sci. 2003;58(2):389–404.

[54] Braithwaite KM, Bowers DG, Nimmo Smith WAM, Graham GW. Controls on floc growth in an energetic tidal channel. J Geophys Res Ocean. 2012;117(2):1–12.

[55] Cross J, Nimmo-Smith WAM, Torres R, Hosegood PJ. Biological controls on resuspension and the relationship between particle size and the Kolmogorov length scale in a shallow coastal sea. Mar Geol. 2013;343:29–38.

[56] Ehrl L, Soos M, Lattuada M. Generation and geometrical analysis of dense clusters with variable fractal dimension. J Phys Chem B. 2009;113(31):10587–99. 10.1021/jp903557m 19594146 10.1021/jp903557m

[57] Lee C, Kramer TA. Prediction of three-dimensional fractal dimensions using the two-dimensional properties of fractal aggregates. Adv Colloid Interface Sci. 2004;112(1–3):49–57. 10.1016/j.cis.2004.07.001 15581554 10.1016/j.cis.2004.07.001

[58] Logan BE, Kilps JR. Fractal dimensions of aggregates formed in different fluid mechanical environments. Water Res. 1995;29(2):443–53.

[59] Spicer PT, Pratsinis SE, Raper J, Amal R, Bushell G, Meesters G. Effect of shear schedule on particle size, density, and structure during flocculation in stirred tanks. Powder Technol. 1998;97(1):26–34.

[60] Edzwald JK, Upchurch JB, O’Melia CR. Coagulation in estuaries. Environ Sci Technol. 1974;8(1):58–63.

[61] Passow U, Alldredge AL, Logan BE. The role of particulate carbohydrate exudates in the flocculation of diatom blooms. Deep Res Part I. 1994;41(2):335–57.

[62] Glasgow LA, Luecke RH. Mechanisms of deaggregation for clay-polymer flocs in turbulent systems. Ind Eng Chem Fundam. 1980;19(2):148–56.

[63] Shanks AL. The abundance, vertical flux, and still-water and apparent sinking rates of marine snow in a shallow coastal water column. Cont Shelf Res. 2002;22(14):2045–64.

[64] Eppley RW, Holmes RW, Strickland JDH. Sinking rates of marine phytoplankton measured with a fluorometer. J Exp Mar Bio Ecol. 1967;1(2):191–208.

[65] Suess E. Particulate organic carbon flux in the oceans—surface productivity and oxygen utilization. Nature. 1980;288(5788):260–3.

